# Ferritin microheterogeneity, subunit composition, functional, and physiological implications

**DOI:** 10.1038/s41598-023-46880-9

**Published:** 2023-11-14

**Authors:** Ayush K. Srivastava, Aliaksandra A. Reutovich, Nathan J. Hunter, Paolo Arosio, Fadi Bou-Abdallah

**Affiliations:** 1 Department of Chemistry, State University of New York, Potsdam, NY 13676 USA; 2 Department of Molecular and Translational Medicine, University of Brescia, 25121 Brescia, Italy

**Keywords:** Biochemistry, Biophysical chemistry, Isoenzymes

## Abstract

Ferritin is a ubiquitous intracellular iron storage protein that plays a crucial role in iron homeostasis. Animal tissue ferritins consist of multiple isoforms (or isoferritins) with different proportions of H and L subunits that contribute to their structural and compositional heterogeneity, and thus physiological functions. Using size exclusion and anion exchange chromatography, capillary isoelectric focusing (cIEF), and SDS-capillary gel electrophoresis (SDS-CGE), we reveal for the first time a significant variation in ferritin subunit composition and isoelectric points, in both recombinant and native ferritins extracted from animal organs. Our results indicate that subunits composition is the main determinant of the mean pI of recombinant ferritin heteropolymers, and that ferritin microheterogeneity is a common property of both natural and recombinant proteins and appears to be an intrinsic feature of the cellular machinery during ferritin expression, regulation, post-translational modifications, and post-subunits assembly. The functional significance and physiological implications of ferritin heterogeneity in terms of iron metabolism, response to oxidative stress, tissue-specific functions, and pathological processes are discussed.

## Introduction

Ferritin is a ubiquitous iron storage protein and a highly conserved supramolecular nanostructure that plays a key role in iron homeostasis^1^. By storing iron safely and reversibly, ferritins overcome the problem of poor iron bioavailability and toxicity while providing an iron source for the synthesis of heme and iron-containing proteins. Human ferritin is composed of two subunit types, named H (for Heavy, ~ 21 kDa) and L (for Light, ~ 19 kDa), which co-assemble in various proportions to form a shell-like protein nanostructure of 24 subunits within which a mineralized iron core is deposited^1–5^. Ferritins in different tissues and organs have different proportion of the two subunits and are referred to as isoferritins; for example, heart, kidney, and brain ferritins are rich in H-chains whereas liver, spleen and bone marrow ferritins are rich in L-chains^1,4,6,7^. The two subunits are encoded by distinct genes located on human chromosomes 11 (for the H-chain) and chromosome 19 (for the L-chain) and have different promoters^1,8^. Although the mechanism through which these subunits co-assemble in vivo remains elusive^9^, the H:L tissue specific ratio is determined by the transcriptional regulation of ferritin genes during development, and in response to iron availability and oxidative stress through the iron responsive element (IRE)/iron response protein (IRP) translational repression system^10^. Briefly, the mRNAs of both subunits contain the IRE structure which can bind the iron responsive proteins IRP1 and IRP2^10^. Under iron deficient conditions, IRP1 and IRP2 bind to the IRE in H- and L-ferritin mRNAs and inhibit their translation^8,11–13^. Conversely, under iron replete conditions iron binds to IRPs and promotes their dissociation from ferritin mRNAs, facilitating their translation^8,11–13^. Whereas tissue-specific distribution of isoferritins have long been recognized^14^, their exact H:L subunits composition, homogeneity and distribution or arrangement around the ferritin shell remain unknown. Microheterogeneity has been observed in isoferritins from diseased tissues revealing more acidic profiles compared to non-diseased tissues, owing to the presence of higher number of H-subunits^15^. Differences in iron content and cell types are unlikely factors for the observed microheterogeneity since chemically reduced apoferritin showed similar heterogeneity profiles than non-treated proteins^16,17^. A number of reasons including surface charge modification of the protein during isolation procedures^10,18,19^, exposed methionine residues at N-terminals^13,20^, and cellular stressors such as changes in cellular iron concentrations and oxidative stress have been proposed^12^. Furthermore, under hypoxic conditions, L-ferritin is more tightly regulated by the IRE/IRP signaling pathway, suggesting that H and L subunits respond independently to cellular iron concentrations^8^.

The large majority of ferritin mechanistic studies have been performed almost exclusively with recombinant human H- or L-homopolymers^1^, or with horse spleen ferritin, that contains only traces of H chains, with only a handful of studies conducted with H-rich or L-rich heteropolymers. This is quite surprising given that natural mammalian ferritins are heteropolymers. One of the impediments to studying ferritin heteropolymers is an inherent difficulty in cloning, expressing, and producing heteropolymer H/L ferritins. Earlier in-vitro reconstitution attempts using chemical denaturation and unfolding of recombinant homopolymers H- and L-ferritins, followed by their renaturation, have largely failed, were irreproducible or unpredictable, or have yielded very low amounts of functional heteropolymer ferritins that are not representative of those occurring naturally^4,6,7,9,21–23^. Nonetheless, the results of these studies have shown that heteropolymer ferritins have properties that differ significantly from homopolymers^5^. To address this shortcoming, we have engineered a novel ferritin expression system to produce recombinant heteropolymer ferritins with different H to L subunit ratios that mimic those found in various organs and tissues ^24^, have characterized their iron uptake and release kinetics, and the structure and morphologies of their iron cores^25–28^. Our unique plasmid design allowed the synthesis of a full spectrum of heteropolymer ferritins, from H-rich to L-rich ferritins and any combinations in-between, enabling us for the first time to examine structural and functional differences of isoferritin populations^24–28^.

**Figure 1:**
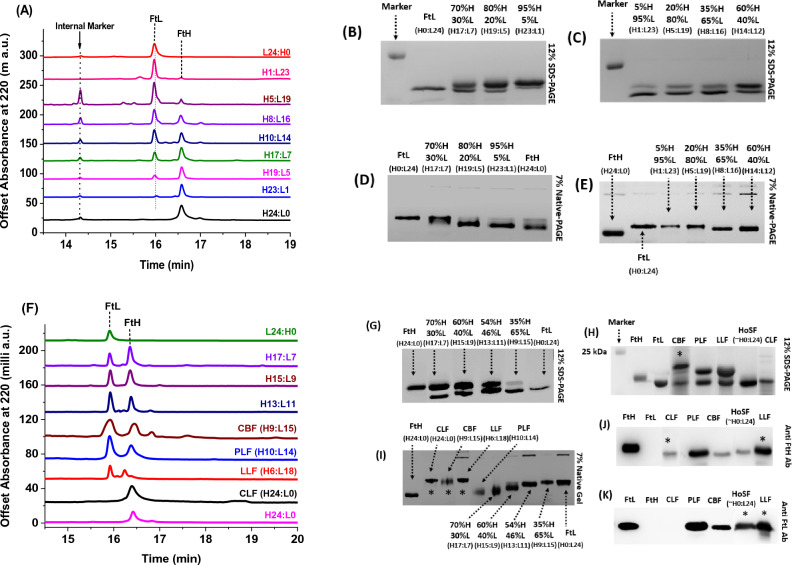
(**A**, **F**) Capillary gel electropherograms (SDS-CGE) of recombinant human homo- (100%L, FtL or L24:H0, and 100%H, FtH or H24:L0) and hetero-polymer (H_x_L_y_) ferritins, cow brain (CBF), pork liver (PLF), lamb liver (LLF), and chicken liver (CLF) ferritins. The ferritin concentrations for the SDS-CGE experiments ranged between 0.5 and 1.0 mg/ml. (**B, C, G, H**) 12% SDS-PAGE and (**D, E, I**) 7% Native-PAGE of the ferritin samples in (**A**). (**J, K**) Western blots using anti-FtH (FtH-Ab) and anti-FtL (FtL-Ab) antibodies. For all gels and Western blots, the protein concentrations varied between 0.5 and 1 μg; FtH, FtL, and horse spleen ferritin (HosF) were used as references. HosF shows a single band because it is mostly L-ferritin (i.e. > 90% L subunits). Gels were stained with Coomassie blue. The gels shown in panels H, I, J and K are combined/grouped gels using other gels ran at different times; the bands that came from different gels are marked with an asterix.

Early studies using purified ferritin from animal organ homogenates subjected to gel or column isoelectric focusing (IEF) showed multiple bands, pointing to the presence of multiple heteropolymers (isoferritins) arising from the combination of different proportions of the H and L subunits within a single tissue^15,18,29,30^. This multiple band pattern or microheterogeneity has been observed within tissue ferritins from humans^15,17,19^, rats^16,31^, and horses^16,17^. However, it has been argued that these multiple bands may be artifacts arising from incomplete equilibrium focusing due to interactions between the proteins, buffer molecules, the ampholytes^14,31–34^, or a methodological artifact arising from discontinuities in the ampholine pH gradients with and without proteins^35^, or due to some subunits being glycosylated^36^. Other studies have shown that the IEF profile of ferritin varies with the commercial ampholyte used during focusing, supporting the view that the discrete bands are artifacts and not distinct isoferritin species^37^. These artifacts have been confirmed in our laboratory (Fig. 1 SI) using recombinant human homopolymer H- and L-ferritins, which consist of one single type of homogenous subunits, and thus should have shown a single migrating band but instead were shown to focus in rather large or multiple bands. All things considered, this controversy over the existence of multiple species of ferritin within the same sample has yet to be resolved, and a better and more accurate method is required to investigate whether the observed charge heterogeneity is due to different subunit composition, isoferritins with different isoelectric points (pI), non-specific interactions between the H and L subunits, a consequence of the cellular machinery producing large multimeric proteins like ferritins, or a result of sheer structural degradation. In this study, we have determined the isoelectric points (pIs) of recombinantly produced ferritin heteropolymers and natural ferritins extracted from different animal organs, determined their H to L subunit ratios, and performed a detailed characterization of their heterogeneity profiles using capillary isoelectric focusing (cIEF), SDS-capillary gel electrophoresis (SDS-CGE), size exclusion and anion exchange chromatography, native and SDS-PAGE electrophoresis, gel isoelectric focusing and immunoblotting. Our results demonstrate that both recombinantly expressed and native animal ferritins display similar microheterogeneity profiles, suggesting a general phenomenon across different species and production methods, and that the underlying mechanisms responsible for ferritin heterogeneity are inherent to the protein itself and its dynamic and complex assembly process rather than being influenced solely by the expression system. Separation of isoferritins can be successfully achieved by sample fractionation using anion exchange chromatography fitted with a diethylaminoethyl (DEAE) cellulose column followed by analysis and quantification of the fractions by capillary gel electrophoresis.

## Materials and methods

### Recombinant ferritin expression and purification

Recombinant human H ferritins were produced in *E. coli* using pET vectors that have a T7 promoter and pAsK vectors that have a Tet promoter with ampicillin resistance. The induction was carried out in LB medium using 0.4 mM IPTG for 4 h at 37 °C. Recombinant human L ferritins were expressed using pDS 20 vectors with ampicillin resistance and transformed into BL21 pLyS strain using M9 minimal medium for 7 h at 37 °C. Recombinant human heteropolymer ferritins with different H to L subunit ratios were produced in *E. coli* Rosetta-gami B strain using a recently engineered pWUR-FtH-TetO-FtL plasmid and different concentrations of inducers, as described in detail elsewhere^24,26^. Transformed cells were induced at 37 °C for 4–6 h using 10–1000 μM isopropyl β-D-1-thiogalactopyranoside (IPTG from Sigma Aldrich), 25–1600 ng/ml of anhydrotetracycline (IBA solutions). The exact concentrations of these inducers depend on the desired H to L ferritin subunit ratio^24^. After centrifugation, the cell pellet was resuspended in 15 ml of 20 mM Tris–HCl pH 7.4, sonicated for 1 min using 3 cycles at 50–60% amplitude, and centrifuged for 10 min at 12,000 rpm. The supernatant was heated at 75 °C, incubated for 10–15 min, centrifuged for 20 min at 12,000 rpm, and then analyzed for protein expression using 12% SDS-PAGE with Coomassie blue (Sigma) staining. The sample was finally subjected to protein precipitation (e.g. 5.3 g of ammonium sulfate was added to 10 ml of supernatant and incubated overnight at 4 °C), followed by an overnight dialysis at 4 °C using at least three 1 L buffer changes of 20 mM Tris HCl, 1mM NaN_3_, pH 7.4. The dialyzed solution was centrifuged at 12,000 rpm for 20 min, to remove any precipitate, and further purified using size exclusion chromatography (SEC) and a 10/600 column packed with ABT 6B resin (Akta Go, GE Healthcare). The SEC column was initially equilibrated with 20 mM MOPS, 100 mM NaCl buffer pH 7.4, and the protein run was performed at a linear flow rate of 0.5 ml/min. The fractions with the highest purity of ferritin were collected and concentrated with 100 kDa cut-off centrifugal devices (Pall) at 4 °C using amicon air pressure concentrator^24^. Protein quantification was performed using the Micro BCA Protein Assay Kit and a Varioskan LUX microplate reader from Thermo Fisher Scientific. Purified recombinant homopolymer and heteropolymer ferritins contained a small iron core (i.e., < 200 ± 50 Fe(III)/ferritin molecule), as determined by an iron reductive mobilization assay^28^.

### Ferritin extraction and purification from animal organs

Animal organs (e.g. pork liver, lamb liver, and cow brain) were obtained from two different slaughterhouses and were exhaustively washed with DI water. Chicken liver was obtained from a local grocery store (Price Chopper, Potsdam, NY). Approximately 100 g of each organ was weighed, mixed with 200 ml of 20 mM Tris buffer pH 7.4, and then blended using a commercial grinder/mixer. The blended sample was transferred into a 50 ml falcon tube, heat treated at 75 °C for 1–2 hrs and centrifuged. The collected soup was concentrated to ~ 5–10 ml using 100 kDa membrane disk filter and a pressurized Amicon ultrafiltration device from EMD Millipore, and then analyzed on a size exclusion chromatography column (ABT 6B resin). We note that the iron content in these native ferritins varied from low (~ 200 Fe(III)/shell for cow brain ferritin) to high (~ 1200 Fe(III)/shell for liver ferritins), depending on the source, the age of the animal, and its overall health. While no attempt was made to explore the effect of iron loading on the pI of these proteins, we found that the pI of an iron loaded recombinant ferritin (100–300 Fe(III)/shell) did not significantly differ from that of an iron-free apo-ferritin that was expressed in an M9 minimal medium without iron, suggesting a minimal effect from a small iron core.

### Native and SDS gel electrophoresis

Native PAGE gels (7% acrylamide) stained with Coomassie blue (Sigma-Aldrich) and SDS − PAGE gels (12% acrylamide) with densitometry analysis (Bio-Rad system) were used to examine the integrity of the ferritin samples and to estimate the ferritin subunit composition from band intensities, respectively.

### Isoelectric focusing (IEF)

Purified human recombinant homo- and hetero-polymer and organ-extracted ferritin samples were initially analyzed for their isoelectric point (pI) by isoelectric focusing (IEF). In these IEF experiments (Fig. SI-1A, SI-1B), 1.0 mm thickness pre-prepared Invitrogen Novex IEF native gels (Thermo Fisher Scientific, Inc.) containing 5% acrylamide and an IEF ampholyte in the 3–7 pH range were used. The precast Novex gel was run on a Mini Gel Tank, XCell SureLock Mini-Cell electrophoresis system at 300 V for 3 h in a cold chamber (2–8 °C). All other reagents (i.e. the IEF anode buffer LC5300, the IEF cathode buffer LC5370, and the IEF pH 3–7 sample loading buffer LC5371) were obtained from Novex by Life Technologies/Thermo Fisher Scientific and were mixed with 2–4 µg protein. After the run was complete, the gels were stained with Coomassie blue dye for visualization.

### SDS-capillary gel electrophoresis (SDS-CGE)

To further examine the integrity of purified ferritin samples and quantify their H to L subunit ratios (both recombinantly produced and animal extracted ferritins), denaturing conditions capillary gel electrophoresis (SDS-CGE) were used. These assays were adopted from Ref.^24^ and used the Sciex SDS-CGE analysis kit which includes an SDS-MW gel buffer (proprietary formulation, pH 8, 0.2% SDS), an SDS-CGE sample buffer (100 mM Tris–HCl pH 9.0, 1% SDS), acidic (0.1 N HCl) and basic (0.1 N NaOH) wash solutions. An Agilent Technologies 50 µm ID bare fused silica capillary with a total length of 33 cm and an effective length of 24.5 cm was used in the SDS-CGE experiments. The SDS-CGE capillary was pre-conditioned under high pressure (i.e. 2.0 bar) using 0.1 N NaOH for 10 min, 0.1 N HCl for 5 min, water for 2 min, and finally a high-pressure flush (4.0 bar) of the SDS gel buffer for 10 min. Prior to any protein run, the capillary was conditioned under high pressure of 4 bar using 0.1 N NaOH (3 min), 0.1 N HCl (1 min), water (1 min) and finally SDS gel buffer (10 min). The protein samples were injected electro-kinetically by applying a negative voltage of—5 kV for 20 s. Protein subunits separation was followed under a negative applied voltage of—16.5 kV for 30 min. A 2.0 bar pressure was applied to both inlet and outlet vials for the duration of the experiment with the capillary temperature maintained at 25.0 °C. The detection wavelength was set at 220 nm (10 nm bandwidth) with a 350 nm reference wavelength (10 nm bandwidth) and a response time of 1.0 s. Typically, ferritin solutions (100 µL at 1–2 mg/ml) were prepared in an SDS sample buffer (> 60% by volume) in the presence of 5 µL 2-beta-mercaptoethanol (5% v/v) from Sigma Aldrich. The ferritin solution was mixed thoroughly, tightly capped and heated in a 100 °C water bath for at least 10 min^24^. The protein solution was then cooled to room temperature prior to running on the 7100 model capillary electrophoresis (CE) instrument from Agilent Technologies.

### Capillary isoelectric focusing (cIEF)

All cIEF experiments were performed on the Agilent Technologies 7100 CE instrument equipped with a UV detector and a 280 nm cutoff filter. The experimental protocol and sample preparation procedure were mainly provided by Agilent Technologies with slight modifications as described below. The temperature settings for the autosampler and the capillary cartridge were 25.0 °C and 10.0 °C, respectively. Unless otherwise noted, all reagents for the cIEF experiments were obtained from Sciex, Corp., and include a neutral coated capillary (part number 477441, 50 µm ID and 67 cm length), or an N-CHO coated capillary, (part number 477601, 50 µm ID, 32 cm working length and 80 cm total length, a cIEF kit, Pharmalyte pI 4.5–7, a 200 mM phosphoric acid (H_3_PO_4_) solution as the anolyte, a 300 mM sodium hydroxide (NaOH) solution as the catholyte, a 350 mM acetic acid (CH_3_COOH) solution as the chemical mobilizer, and a 4.3 M urea solution as the capillary cleaning solution. The urea-containing cIEF polymer solution was prepared by dissolving urea at a final concentration of 3.75 M into the sciex’s cIEF polymer solution. The sacrificial ampholytes for the anodic and cathodic blockers were iminodiacetic acid (IDA) and L-arginine (Arg), respectively. All ferritin samples were initially dissolved in 20 mM Tris–HCl, pH 7 buffer solution. Protein samples (5–10 mg/ml) before cIEF analysis were prepared in a cIEF master mix containing 200 μL of 3.75 M urea-cIEF gel, 12 μL or 1.8% w/v Pharmalyte pI 4.5 to 7, 500 mM Arg, 200 mM IDA and 2 μL of the pI 7.0 synthetic peptide marker used an internal standard. Before the experiment, the neutral capillary was conditioned by rinsing at 3.5 bar with water for 5 min, then with 350 mM acetic acid for 15 min, and with cIEF gel for 5 min. The capillary was then rinsed for 10 min at 3.5 bar with a 4.3 M urea capillary cleaning solution to remove any proteins that might have adhered to the internal capillary walls. The sample mixture was introduced into the capillary by rinsing it for 180 s at 2 bar. Focusing was carried out at a field strength of 25 kV in reverse polarity with 200 mM phosphoric acid as the anolyte, and 300 mM sodium hydroxide as the catholyte. The focused peaks were chemically mobilized across the detection window by replacing the catholyte vial with a 350 mM acetic acid solution and then applying a field strength of 30 kV in reverse polarity. The focused protein bands were detected by absorbance at 280 nm. To construct a calibration curve, a cIEF polymer solution was prepared separately using 2 μL of pI 7.0, 5.5 and 4.1 synthetic peptide markers.

### Immunoblotting

Purified human ferritin (1 µg) was loaded onto 7.5% native PAGE and transferred to Hybond-P Membrane (GE) using western blot. The immunoblotting primary antibodies for the human H- and the human L-ferritin subunits were Biomatik CAU30392 and Biomatik CAU30796 monoclonal antibodies, respectively. After incubation with horseradish peroxidase HRP-conjugated secondary antibody from Rockland (610–4302) anti-mouse HRP antibody generated in rabbit, membranes were developed with Super Signal West Pico Chemiluminescent Substrate (Biorad) and visualized on an AZI280 imaging system from Azure Biosystems Inc.

### DEAE anion-exchange chromatography

The size exclusion chromatography-purified protein was applied to a DEAE column (25 ml Macro-Prep DEAE, 50 μm particle size, 10 ml column volume, Catalog no 1580020 from Bio-Rad Laboratories, Inc.) equilibrated with 20 mM Tris buffer, pH 7.4. The elution buffer was 20mM Tris, 500 mM NaCl, pH 7.4. Both continuous salt (gradient) and stepwise (isocratic) elution have been performed, with NaCl concentrations indicated on Fig. 4. The eluate was continuously monitored at 280 nm for protein detection, and fractions of 2 ml were collected at a flow rate of 1 ml/min.

## Results

### Ferritin integrity and subunit composition

To investigate the integrity of heteropolymer ferritins and their H and L subunit composition, SDS and native-PAGE experiments and capillary gel electrophoresis under denaturing conditions (SDS-CGE) were performed. Samples from different sources were used and included human recombinant heteropolymer ferritins expressed in *E. coli* using our newly engineered plasmid design^24^, and native ferritins extracted from different animal organs (chicken, lamb, and pork livers, and cow brain) using established purification protocols^14^. For human recombinant heteropolymer ferritins, the pWUR-FtH-TetO-FtL transformed *E. coli* cells (i.e., Rosetta-gami B strains) generated isoferritins with different H to L subunit ratios, the composition of which depended on the concentrations of IPTG and Tet, the two inducers that control the expression of H and L subunits, respectively^24^. For native ferritins, the animal organs were sliced, thoroughly washed with DI water, and then blended with 20 mM Tris pH 7.4 buffer until a smooth suspension is obtained^14^. The suspension was centrifuged, and the supernatant was heat-treated at 75 °C for 1–2 hrs to separate ferritin from most other proteins and cellular components present in the sample.

Figure 1A, F show the SDS-CGE electropherograms of different ferritin samples with well-resolved peaks of the L- and H-subunits. From the area under the CGE peaks, the subunit composition of heteropolymer ferritins was calculated with the percent composition of H and L subunits displayed next to each electropherogram. While native ferritins extracted from the livers and brains of pork, lamb, and cow showed a mixture of H and L subunits (i.e. heteropolymer ferritins), chicken liver ferritin exhibited one single peak that eluted at the same time as recombinant human H-homopolymer, in accord with an earlier study^38^. As expected, all heteropolymer and native ferritins (except chicken liver ferritin) showed two bands on a 12% SDS PAGE, corresponding to H and L subunits (Fig. 1B, C, G, H) with the gel band intensities matching the H:L subunit ratio determined by SDS-CGE. Western blot analysis using anti-ferritin heavy chain (Fig. 1J) and anti-ferritin light chain (Fig. 1K) antibodies confirm the presence of ferritin H and L chains, respectively. The reason that horse spleen ferritin (HosF) shows a single band, despite it being a heteropolymer protein is because it is mostly L-ferritin with only 1 or 2 H-subunits (i.e. > 90%L subunits). As expected, the 7% native-PAGE showed single bands for all ferritin samples examined (Fig. 1D, E, I), the mobility of which depended on their H to L subunit composition. The observed single bands on native-PAGE suggest that the H- and L-subunits have co-assembled into one type of ferritin species, while the different band mobilities suggest differences in the proteins’ pI values. Notably, pork liver ferritin (PLF) ran closer to recombinant human H-ferritin (FtH), whereas chicken liver (CLF), lamb liver (LLF), and cow brain (CBF) ferritins ran closer to human L-ferritin, suggesting that the pI of PLF is more acidic compared to CLF, LLF, and CBF, even though CLF is purely an H-ferritin (Fig. 1I). The reasons for these discrepancies could be due to different protein sequences, posttranslational modifications (i.e. glycosylation, phosphorylation, ubiquitination, methylation, acetylation, lipidation, proteolysis, etc.) or other structural variations affecting the sidechain pKa that could have modified the net charge on the ferritin surface, whether directly after protein synthesis or over the lifetime of the protein (i.e. 2–3 days after synthesis)^18,35,39,40^.

### Determination of ferritin isoelectric points (pI)

Capillary isoelectric focusing (cIEF) is a true capillary electrophoresis protein identification technique that provides pI information on a complex mixture of protein isoforms. While both conventional gel-based isoelectric focusing (IEF) and cIEF methods separate charged analytes based on their isoelectric points (pI) under the influence of an electric field, gel-related issues in IEF such as variations in gel composition, pH gradients, buffer conditions, band broadening and mixing due to diffusion effects can result in artifacts and distorted or smeared peaks, as discussed in the introduction section. Unlike IEF (Fig. SI-1), methodological artifacts in cIEF are generally minimized or even avoided since the separation takes place within a solid and stable medium inside a narrow capillary which allows for sharper and better-resolved peaks.

**Figure 2:**
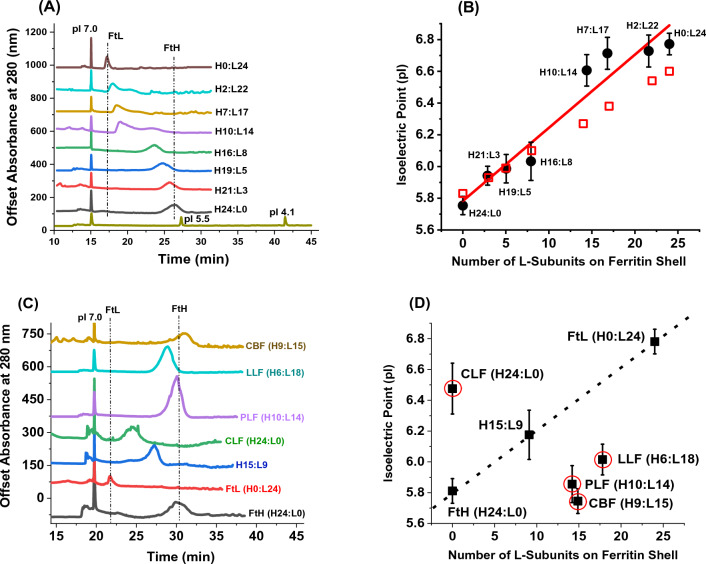
(**A, C**) Representative cIEF separation of recombinant human homo- and heteropolymer ferritins (~ 5–10 μg/μL) and native ferritins from animal organs (~ 5–10 μg/μL), with pI 7 as an internal marker. CBF, LLF, PLF, and CLF are cow brain, lamb liver, pork liver and chicken liver ferritins, respectively. (**B, D**) Plot of the isoelectric point (pI) as a function of the number of L-subunits present on the ferritin shell. The red squares on panel B represent the computed theoretical pI values of all recombinant human ferritins employed in this study. The red circles on panel D are to indicate that native ferritins do not follow a linear relationship as recombinant ferritin. A calibration curve was performed with three Sciex standards and pI values of 7.0, 5.5 and 4.1. Samples were focused for 12 min at 25 kV using reverse polarity and mobilized for 30 min at 30 kV using reverse polarity with 350 mM acetic acid as the chemical mobilizer. The error bars represent a standard deviation from at least 2 runs per sample.

Figure 2A, C show the electropherograms of different ferritin samples with a pI 7 marker used as an internal standard. Heteropolymer ferritins with the highest L-subunit content eluted first and those with higher H-subunit content progressively moved towards the H-homopolymer and eluted last. The pI of homopolymer L-ferritin (FtL) was the closest to the pI 7 marker while that of H-ferritin (FtH) was closer to the pI 5.5 marker. Using a standard calibration curve with three known pI markers (7.0, 5.5, and 4.1), the pIs of heteropolymer ferritins were determined and displayed good linearity when plotted against the number of L-subunits present on the protein shell (Fig. 2B). L-rich heteropolymers revealed more basic pI values (6.6 < pI < 6.8) compared to H-rich heteropolymers (5.8 < pI < 6.0). Although the pIs of recombinant human ferritins are, to our knowledge, reported here for the first time, they markedly differ from those of native animal ferritins determined earlier using slab gel IEF^15–19,29–31^. For instance, chicken liver ferritin (i.e. an exclusively H homopolymer ferritin) is less acidic than human H ferritin, while cow brain and pork liver ferritins (~ 40%H and 60% L content) and lamb liver ferritin (~ 25%H and 75% L) are more acidic than their corresponding human heteropolymer ferritins with similar H and L composition (Figs. 1F, 2D). The pI difference between recombinant and natural human ferritins is partially attributed to the N-terminus that is blocked in the native and free in the recombinant ferritins^41^. Interestingly, a linear plot of the melting temperature (Tm) vs. L-subunits was recently reported^28^, suggesting that ferritin L-subunits exhibit similar effects on the proteins’ thermostability and their isoelectric points. Remarkably, out of the available ferritin structures, an improved web-based visualization tool (PDB2PQR) of the Adaptive Poisson-Boltzmann Solver software^42^ provided pI values that perfectly matched our experimental values (i.e. 5.83 for human H-ferritin, PDB ID 7A6A; 6.60 for human L-ferritin, PDB ID 6WX6; and 5.95 for cow ferritin, PDB ID 7U5L). Using the above, the theoretical pI values of all recombinant human ferritins employed in this study were computed and displayed as red squares in Fig. 2B.

Although the genomes of many species contain multiple copies of ferritin H and ferritin L sequences, the chicken genome contains only a single copy of the H-subunit gene, which encodes a 179 amino acid protein with a calculated molecular weight of ~ 21 kDa and a pI of 6.1, similarly to chicken spleen ferritin which has a pI of 6.14^43^. The H-subunit of chicken ferritin has a 93% amino acid sequence identity to the human ferritin H-subunit, and chicken liver ferritin (CLF) is similar in composition to human mitochondrial ferritin in that both types of ferritin are H-homopolymers. However, in the absence of comparative biochemical, physiological, and evolutionary studies, it would be premature to discuss the reasons for the unique composition of CLF compared to that of other liver ferritins discussed here, which could include evolutionary lineage, different gene regulation mechanisms, or the need for a unique metabolic or physiological demand for avian. For instance, a homopolymer H-ferritin in chicken liver may suggest a heightened need for rapid iron sequestration, possibly related to the metabolic demands or specific physiological functions of avian livers. On the other hand, the unique composition of chicken liver ferritin might have implications in avian health or in resistance to diseases.

It is worth noting that the broadening of the cIEF peaks observed in Fig. 2A is likely a combination of protein adsorption due to electrostatic interactions with the capillary wall, protein aggregation, molecular diffusion, and/or slow mass transfer caused by convection or interactions of the large size and heat stable ferritin molecules with the capillary surface, or the presence of multiple protein species (isoforms or isoferritins) with slightly differing pI values. Additionally, to our knowledge this is the first time that cIEF experiments have ever been attempted on excessively large proteins like ferritin.

### Capillary gel electrophoresis (CGE) of ferritin samples following fractionation by size exclusion chromatography (SEC)

**Figure 3:**
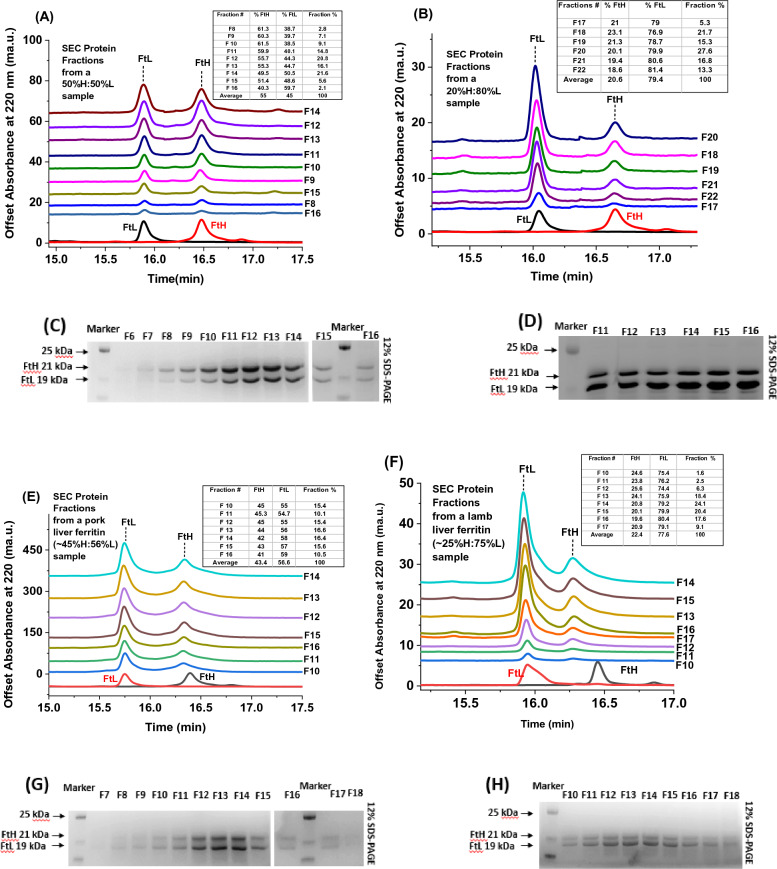
(**A, B, E, F**) Representative SDS-CGE electropherograms of SEC-fractionated samples of recombinant human homo- and hetero-polymer ferritin and lamb and pork liver ferritin following size exclusion chromatography using either 6% B agarose beads (50–150 µm) or ABT 6B resin. (**C, D, G, H**) 12% SDS-PAGE of the collected fractions.

To verify the degree of ferritin microheterogeneity and quantify the H and L subunit composition, recombinant and native ferritin samples were fractionated on size exclusion chromatography and analyzed with capillary gel electrophoresis under denaturing conditions (SDS-CGE). The SDS-CGE electropherograms of two ferritin samples (~ 50%H:50%L and ~ 20%H:80%L) showed well-resolved peaks for the H- and L-subunits, with migration times difference of 0.6–0.7 min between the two subunits (Fig. 3A, B). From the area under the CGE peaks, the subunit composition of the different protein fractions was determined and showed average values of (55 ± 7) %H subunits and (45 ± 7) %L subunits for the first sample (Fig. 3A, i.e. 13 ± 1 H-subunits and 11 ± 1 L-subunits), and (20.6 ± 1.6) %H subunits and (79.4 ± 1.6) %L subunits for the second sample (Fig. 3B, i.e. 5 ± 0.1 H-subunits and 19 ± 0.3 L-subunits). The SEC results of both recombinant and native preparations showed that the early fractions were H-chain richer than the tail fractions (Table 1), suggesting a similar subunit heterogeneity irrespective of the ferritin source. This SEC separation is based on molecular size and does not account for small variations due to structural changes or differences in pI values. Additional results for other L-rich and H-rich ferritins are shown in Figures SI-2. Table 1 displays a more detailed analysis of subunit composition and percent distribution of the collected fractions and shows that the L-rich ferritin sample (i.e. ~ 20%H:80%L) has narrower distribution compared to the 50:50 mixed H:L sample (i.e. ~ 50%H:50%L). All fractions were also examined on 12% SDS-PAGE and showed band intensities in line with the CE-CGE data (Fig. 3C, D). A similar analysis was performed on ferritin samples extracted from lamb and pork livers and showed similar results, although with narrower distribution and much less variations in the H and L subunit composition (Fig. 3E–H).

**Table 1:** H to L subunit ratios of the different fractions collected by SEC for recombinant human heteropolymer ferritins (samples 1 and 2), and lamb and pork liver ferritins (samples 3 and 4) and their percentage abundance.

Sample 1: recombinant human H:L heteropolymer ferritin (~ 55%H:45%L)	%H:%L ratio SEC-fractionation	% Abundance	Sample 2: recombinant human L-rich heteropolymer ferritin (~ 20%H:80%L)	%H:%L ratio SEC-fractionation	% Abundance
Fraction 8	61:39	2.8%	Fraction 17	21:79	5.3%
Fraction 9	60:40	7.2%	Fraction 19	21:79	15.3%
Fraction 10	61:39	9%	Fraction 18	23:77	21.7%
Fraction 11	60:40	14.7%	Fraction 20	20:80	27.6%
Fraction 12	56:44	21%	Fraction 21	19:81	16.8%
Fraction 13	55:45	16%	Fraction 22	18:82	13.2%
Fraction 14	50:50	21.7%			
Fraction 15	51:49	5.6%			
Fraction 16	40:60	2%			
Weighted Average	55:45	100%	Weighted Average	21:79	100%

Sample 3: ferritin extracted from lamb liver (~ 25%H:75%L)	%H:%L ratio SEC-fractionation	% Abundance	Sample 4: ferritin extracted from pork liver (~ 45%H:55%L)	%H:%L ratio SEC-fractionation	% Abundance
Fraction 10	25:75	1.6%	Fraction 10	45:55	24.5%
Fraction 11	24:76	2.5%	Fraction 11	45:55	10.1%
Fraction 13	24:76	18.5%	Fraction 13	44:56	46.7%
Fraction 12	26:74	6.3%	Fraction 14	42:58	16.4%
Fraction 14	21:79	24.1%	Fraction 15	43:57	15.6%
Fraction 15	20:80	20.3%	Fraction 16	41:59	10.4%
Fraction 16	20:80	17.6%	Fraction 17	42:58	6.2%
Fraction 17	21:79	9.1%			
Weighted Average	21.5:78.5	100%	Weighted Average	43:57	100%

### Capillary gel electrophoresis (CGE) of ferritin samples following fractionation using diethylaminoethyl (DEAE) cellulose column

**Figure 4:**
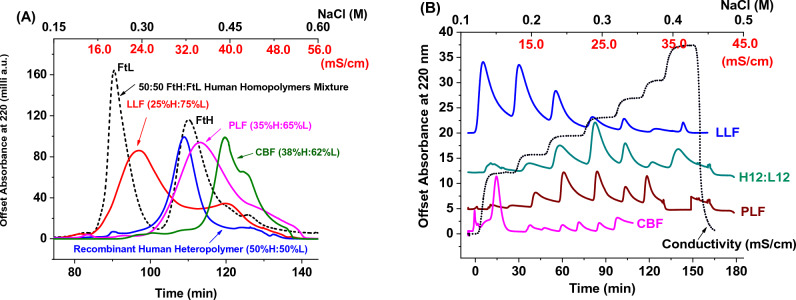
Overlay of DEAE-cellulose chromatograms of recombinant human ferritin samples and native ferritin extracted from animal organs. DEAE-gradient elution (**A**) and isocratic or stepwise elution (**B**). The top X-axes represent the conductivity of the solution at various NaCl concentrations.

To further investigate sample microheterogeneity, we performed two types of ion-exchange chromatography (i.e. continuous salt gradient elution, and stepwise or isocratic elution with fixed and increasing concentrations of NaCl) using diethylaminoethyl (DEAE) cellulose column (Bio-Rad Laboratories, Inc.). We note that the DEAE columns are weak anion exchangers able to resolve complex biological mixtures including proteins with slightly different charges. SEC-purified ferritin samples were loaded onto a 25 ml DEAE column equilibrated with 20 mM Tris buffer, pH 7.4, and seven 2 ml fractions were eluted (F15-F22) using continuous salt gradients (Fig. 4A). These fractions were then pooled and re-loaded onto the same column but this time four 2 ml fractions were collected (F2-F5) using stepwise or isocratic elution (Fig. 4B).

**Figure 5:**
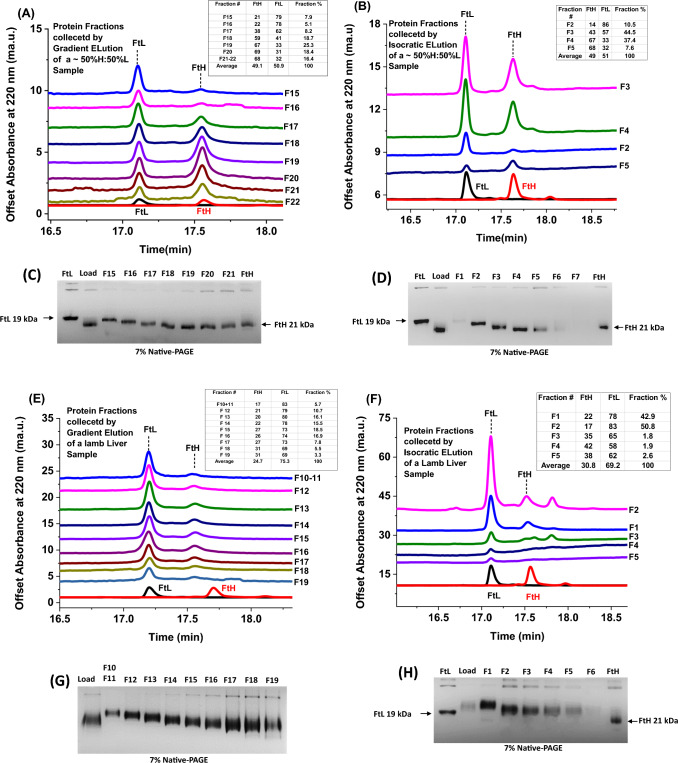

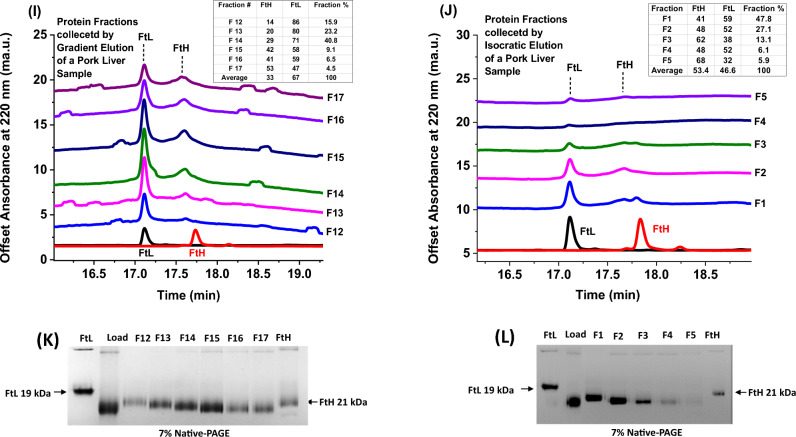
SDS-CGE electropherograms of fractionated samples obtained by DEAE gradient and isocratic elution methods of (**A, B**) recombinant human heteropolymer ferritin sample (~ 50%H:50%L), (**E,F**) lamb liver ferritin, and (**I, J**) pork liver ferritin. (**C, D, G, H, K, L**) 7% Native-PAGE of the different fractions collected. The “load” lane is that of the pooled fractions.

All fractions collected via the gradient and isocratic elution methods were then analyzed by native-PAGE and SDS-CGE to quantify their H and L subunit ratios (Fig. 5A–L). The results showed a much wider distribution of isoferritin species, compared to the SEC-fractionation method, and a larger variation in the H and L subunit content, with the early fractions being strongly enriched in the L-chain compared to the late fractions, both in the recombinant and native ferritins. Thus, gradient elution of ferritin samples is an effective method to separate isoferritin species based on pI values and provides a more detailed profile of subunit distribution than isocratic elution. Notably, native-PAGE results of all collected fractions showed a clear separation between the different isoferritin species, whereby the H-rich ferritins migrate closer to the more acidic homopolymer H-ferritin and those with higher L-subunit content migrating closer to the more basic homopolymer L-ferritin (Fig. 5C, D). Furthermore, lamb and pork liver ferritins (i.e. pooled fractions, 2nd lane on native-PAGE gels, Fig. 5H, L) migrated very differently than recombinant human ferritin, with lamb liver ferritin migrating higher than human L-homopolymer ferritin (FtL), and pork liver ferritin migrating lower than human H-homopolymer ferritin (FtH). The different surface charges and pI values of the native ferritins compared to recombinant human ferritins are attributed to their different amino acid sequences.

## Discussion

While slab gel isoelectric focusing (IEF) has been central for studying ferritin subunit composition and heterogeneity, it has its limitations and potential artifacts^15–19,29–31^. Here, we have used capillary isoelectric focusing (cIEF) and developed a robust method based on ion-exchange chromatography combined with capillary gel electrophoresis to characterize ferritin heterogeneity more accurately, both recombinantly produced and native ferritins extracted from animal organs. We chose to use capillary isoelectric focusing (cIEF) because it is a sensitive and powerful analytical method that can separate proteins and peptides according to differences in their isoelectric points (pI) and has been routinely used to quantify and assess the purity of biological molecules, extent of degradation and microheterogeneity. Briefly, the cIEF separation process involves multiple steps and uses several reagents including ampholytes (to generate pH gradient and resolve protein mixtures), pI markers (to calculate the pI of molecular species present in the sample), cathodic stabilizer (to force the pH gradient to form before the capillary detection window), and anodic stabilizer (to minimize the loss of ampholytes into the anolyte). Sample focusing occurs inside a neutral-coated capillary whereby a pH gradient is formed once a voltage is applied, with one capillary end immersed in the low pH anolyte solution and the other capillary end is in the high pH catholyte solution. Separation occurs during focusing where different charged species migrate to pH zones equal to their pI values where they become neutral. The separated species and the ampholytes present in the sample are then chemically mobilized with acetic acid across the capillary window for protein detection at 280 nm^44^.

Our results show that the mean pI of recombinant human ferritins detected by cIEF is correlated with their subunit composition, confirming that the H:L ratio is a major determinant in the ferritin heterogeneity. The fractionation on SEC and DEAE columns was performed to explore the microheterogeneity of the ferritin preparations, in particular to verify possible differences between the native and the recombinant heteropolymers. While SEC fractionation was minor, it nonetheless showed that the early fractions were richer in H chains than the late fractions, both in recombinant and native ferritins, in accord with the H chains being slightly larger than the L chains. More effective fractionation was observed with a DEAE column using salt gradient, showing a major enrichment of L-chains in the early fractions, both in native and recombinant ferritins. This indicates that microheterogeneity is also a property of recombinant ferritins that is associated with isoferritins of different H:L composition. It is presently unclear if other factors may contribute to the microheterogeneity of recombinant ferritins, but posttranslational modifications such as glycosylation are excluded since these changes do not occur with recombinant proteins.

It has been long recognized that tissue ferritins are not homopolymer proteins but families of hybrid molecules consisting of isoferritins of different proportions of H and L subunits^14–19^. In humans, there exist three functional ferritin genes that encode three types of chains, the heavy chain (H-chain, FtH, 183 amino acids long), the light chain (L-chain, FtL, 175 amino acids long), and an H-like chain that makes up the 24 homopolymeric mitochondrial ferritin^45,46^. The cytosolic H- and L-chains are under the same iron-dependent post-transcriptional control and co-assemble in different proportions into 24-subunit heteropolymers giving rise to a large number of isoferritins with a specific tissue distribution^4,47^. Despite its limitation, experiments using gel electrofocusing and ferritins from rat, horse, and human displayed a consistent pattern of heterogeneity and a broad spectrum of isoferritins with pI values ranging from 4.8 to 5.8 for human^15,17,19^, 5.1 to 5.9 for rats^16,31^, and 4.1 to 5.1 for horse^16,17^. Heart isoferritins from humans and rats were found to be more acidic than the corresponding liver ferritins, whereas isoferritins from horse heart were more basic than those from horse liver. Although considerable variations within the same species could occur between individuals in different physiological states, over a dozen isoferritins have been detected in the organs of these animals corresponding to the two known major subunits (i.e. ~ 19 kDa for L-subunit and 21 kDa for H-subunit)^15–17,19,31^. It is quite possible that ferritin heterogeneity may be an inherent characteristic of the protein and a result of its assembly process. Alternatively, ferritin’s net charge and structural heterogeneity may be a consequence of the cellular machinery producing recombinant proteins. This is particularly important in the case of large and complex molecules with high molecular weight like ferritins (i.e. ~ 500 kDa) where posttranslational modifications during synthesis, and/or degradation during purification or storage could lead to surface charge modifications and thus structural variations and heterogeneity.

Although the cellular assembly mechanism of ferritin subunits remains elusive, it is tempting to discuss possible reasons for the observed heterogeneity and the physiological implications of isoferritins distribution in tissues, even though our speculation is partly derived from in-vitro results of ferritin subunits assembly, and the realization that this process may be completely different in-vivo that could involve protein chaperones, other biomolecules, or cellular components. First off, the preferential formation of H/L hybrid heteropolymers from the denaturation of H- and L-homopolymers followed by their renaturation at neutral pH^9^ explains why H- and L-homopolymers are poorly populated in mammalian tissues, suggesting a recognition specificity between H and L subunits and the preferential formation of H/L heterodimers over H–H or L-L homodimers^5^. A completely random interaction between H and L subunits is unlikely since this should lead to the formation of a full spectrum of isoferritin species from H24:L0 to H0:L24 and anything in between. In this work, L-rich lamb liver and pork liver ferritins (average composition of ~ 25%H:75%L and ~ 35%H:65%L, respectively) showed a distribution of H and L subunits ranging between ~ 20–40%H and ~ 80–60%L for lamb and ~ 15–50%H and ~ 85–50%L for pork using both gradient and isocratic methods of fractionation and elution (Table 2). However, the majority of species (i.e. 85–95% in the case of lamb liver, and 80–90% in the case of pork liver) consist of isoferritins with H and L composition close to the average composition of these ferritin types (Table 2). Similarly, the percent distribution of recombinant human heteropolymer ferritin is largely centered around the average composition of ~ 50%H:50%L with only about 12% of species containing 15–20% H subunits (Table 2). These results suggest that the interactions between H and L subunits in heteropolymer ferritins are more specific than random, with up to ~ 20% of species deviating from the calculated weighted average majority.

**Table 2:** H to L subunit ratios of the different fractions collected by ion exchange chromatography using DEAE column (IEX-DEAE) and gradient or isocratic elution for recombinant human heteropolymer ferritins (samples 1 and 2), lamb liver ferritins (samples 3 and 4) and pork liver ferritins (sample 5) and their percent abundance.

Sample 1: recombinant human H:L heteropolymer (~ 50%H:50%L) (gradient elution)	%H:%L ratio IEX-DEAE fractionation (gradient elution)	% Abundance	Sample 1: recombinant human H:L heteropolymer ferritin (~ 50%H:50%L) (isocratic elution)	%H:%L ratio IEX-DEAE fractionation (isocratic elution)	% Abundance
Fraction 15	21:79	7.9%	Fraction 2	14:86	10.5%
Fraction 16	22:78	5.3%	Fraction 3	43:57	44.5%
Fraction 17	38:62	9%	Fraction 4	67:33	37.5%
Fraction 18	59:41	22.8%	Fraction 5	68:32	7.5%
Fractions 19	67:33	31.8%			
Fraction 20	69:31	16.4%			
Fraction 21	68:32	6.7%			
Weighted Average	57:43	100%	Weighted Average	51:49	100%

Sample 3: ferritin extracted from lamb liver (~ 25%H:75%L) (gradient elution)	%H:%L ratio IEX-DEAE fractionation (gradient elution)	% Abundance	Sample 3: ferritin extracted from lamb liver (~ 25%H:75%L) (isocratic elution)	%H:%L ratio IEX-DEAE fractionation (isocratic elution)	% Abundance
Fractions 10, 11	17:83	5.7%	Fraction 2	20:80	50.7%
Fractions 12,13	21:79	26.8%	Fraction 1	22:78	42.8%
Fraction 14	22:78	15.5%	Fraction 3	35:65	1.9%
Fraction 15	27:73	18.6%	Fraction 4	38:62	2%
Fraction 16	26:74	17%	Fraction 5	42:58	2.6%
Fraction 17	27:73	7.8%			
Fractions 18, 19	31:69	8.7%			
Weighted Average	24:76	100%	Weighted Average	22:78	100%

Sample 4: ferritin extracted from pork liver (~ 35%H:65%L) (gradient elution)	%H:%L ratio IEX-DEAE fractionation (gradient elution)	% Abundance	Sample 4: ferritin extracted from pork liver (~ 35%H:65%L) (isocratic elution)	%H:%L ratio IEX-DEAE fractionation (isocratic elution)	% Abundance
Fraction 12	14:86	16%	Fraction 1	24:76	13%
Fraction 13	20:80	23.1%	Fraction 2,4	28:72	56%
Fraction 14	29:71	40.8%	Fraction 3	36:64	25%
Fraction 15	42:58	9.1%	Fractions 5	40:60	6%
Fraction 14	41:59	6.5%			
Fraction 17	53:47	4.5%			
Weighted Average	27.5:72.5	100%	Weighted Average	30:70	100%

Whilst the exact biological functions of isoferritins remain largely obscure, their distribution in tissues appear to correlate with ferritin activities. By producing ferritins with distinct subunit compositions, tissues and cells can tailor their iron storage capacity and antioxidant properties according to their specific physiological requirements. For instance, L-rich ferritins exhibit slow iron uptake kinetics and are typically found in tissues that store large amount of iron such as liver and spleen, whereas H-rich ferritins have fast iron uptake kinetics and are found in active tissues that do not have iron storage function but are efficient in iron detoxification and cellular protection such as heart, kidney, brain, and erythroid cells^1,4,11,19,47^. However, because tissues like liver, spleen, and bone marrow have high iron storage needs, it is not unrealistic for their cells to produce ferritins with a higher proportion of H subunits, since H-rich ferritins have a high iron uptake capacity and could quickly respond to a burst of iron influx, thus keeping the cell protected from iron-induced oxidative damage (i.e. the in-vivo iron storage and mineralization process may require the oxidative activity of H-subunits). In contrast, tissues with lower iron storage requirements such as heart, kidney, and brain may produce isoferritins with a high proportion of L subunits that could safely store large amounts of iron for later use. For instance, it has been shown that human heart ferritin forms a discrete iron core with an iron content > 1000 Fe/shell, suggesting that even if L subunits are not dominant in a given organ or tissue, the ferritin can still store a considerable load of iron^48^. Additionally, since H- or H-rich ferritins are known to possess stronger antioxidant activity compared to L- or L-rich ferritins^49^, it may be expected for both types of ferritins to co-exist in cells. Thus, the production of isoferritins with distinct subunit compositions allows different tissues and cells to adapt to their microenvironments, maintain cellular iron balance, protect against oxidative damage, and support tissue-specific functions.

The relationship between H-ferritin, autophagy, and iron release is an emerging area of research, but the precise mechanism of how H-ferritin triggers autophagy is currently not known. In vivo, iron loading into ferritin is mediated by the iron chaperone protein PCBP1 with the assistance of glutathione-bound ferrous ions^50^. Following iron loading, ferritin is eventually moved to the lysosomal compartment via ferritinophagy, a process that depends on the interaction of nuclear receptor coactivator-4 (NCOA4) with H-ferritin^51,52^. NCOA4 appears to self-oligomerize through its N-terminal coiled-coil domain followed by binding to H-ferritin via an intrinsically disordered region to form ferritin–NCOA4 particles. These particles are then recognized by the autophagy adaptor Tax1 binding protein 1 (TAX1BP1) which will then be processed by the macroautophagy and endosomal microautophagy machineries^53^. This autophagic degradation of ferritin is mainly controlled by the amount of NCOA4 in the cell, depending on cellular iron levels. In the case of iron overload, NCOA4 binding to FTH1 is inhibited by HERC2 binding to NCOA4 followed by its proteasomal degradation. However, it is still unknown whether the ferritin autophagic degradation process is the only mechanism by which iron is released from the protein or whether other auxiliary iron mobilization mechanisms that provide an immediate and more economical source of iron to cells exist. A recent study from our laboratory suggests that in addition to the generally accepted iron mobilization mechanism through ferritin proteolytic degradation, there exists an auxiliary iron reductive mechanism that utilizes long-range electron transfer pathways, facilitated by the ferritin shell^25^. Interestingly, deletion of the H-ferritin gene (but not the L-ferritin gene) leads to embryonic lethality in mice, a deregulation of iron metabolism, oxidative stress, inflammation, and multi-organ damage, suggesting that H-ferritin is indispensable for proper iron homeostasis^54–56^.

To our knowledge, there are no clear evidence that homopolymer L-ferritins exist in tissues despite the fact that an old study has hinted to that possibility more than four decades ago^57^. Although NCOA4 has been shown to interact exclusively with H ferritin and not with L ferritin, it is unclear how the subunit composition of ferritin will affect this interaction or whether a homopolymer L ferritin or an L-rich ferritin that is loaded with iron is degraded via the same mechanism (i.e. via lysosomal degradation). Furthermore, we do not know whether a higher proportion of H subunits in a ferritin molecule would support a “faster turnover” under conditions of iron deficiency, nor do we fully understand how the NCOA4-ferritin condensate forms. Moreover, it is unclear whether the size of the iron core inside ferritin affects its interactions with NCOA4, or what role, if any, does the ferritin L subunit play in the recognition event and the NCOA4-mediated incorporation of ferritin into cytosolic condensates. We speculate that the ferritin subunit composition could affect the nature of the ferritin-NCOA4 complex or condensate, the trafficking route of the complex, and/or the efficiency of removal of the condensates from the lysosome. It is also possible that other mechanisms or processes of iron retrieval from L or L-rich ferritins exist but are not yet discovered. For more information on this topic, we direct the reader to an excellent review on the mechanisms controlling cellular and systemic iron homeostasis^58^.

In an earlier study^27^, we showed that L-rich ferritins exhibit spherical and large iron cores with an average diameter of ~ 5.9 nm, whereas H-rich ferritins present elongated, dumbbell, and crescent-shaped cores with an average core diameter of ~ 3.7 nm, suggesting a significant correlation between protein shell composition, iron core shape, and likely biological function^1–5,59^. Such structural differences may offer unique characteristics to isoferritins and contribute to the functional diversity of ferritins in terms of iron accessibility to cells and protection against oxidative stress. They may also affect and influence the interactions of ferritins with other protein partners and have important implications on many physiological processes including intracellular signaling and the pathogenesis of diseases. For instance, we have found that the smaller and elongated iron core diameter in H-rich ferritins exhibit a higher degree of crystallinity compared with L-rich cores which appear more diffuse and amorphous^27^. The less crystalline and low mineral order of L-rich ferritins iron cores is suggested to facilitate rapid iron turnover in support of the physiological role of these types of protein as a general iron source^47^. We postulate that aberrant ferritin heterogeneity or abnormal changes in tissue ferritin subunit composition can disrupt the balance of iron homeostasis, alter the protein’s antioxidant capacity, and/or impair the normal functioning of cells and tissues. In fact, gene deletions, genetic mutations, or altered H or L gene expression levels have been implicated in a variety of diseases^4,10,45–47,60,61^. Thus, understanding ferritin heterogeneity could shed light on the complex roles of ferritin in maintaining iron homeostasis, cellular function, and overall health, and may offer new avenues for developing diagnostic tools and therapeutic interventions for a host of disorders, including inflammatory conditions, cancer, and neurodegenerative diseases^62,63^.

### Supplementary Information

Supplementary Information 1.

Supplementary Information 2.

## Data Availability

The datasets generated during and/or analyzed during the current study are available from the corresponding author on reasonable request.
