# Supplementary material for: Ferritin microheterogeneity, subunit composition, functional, and physiological implications
Source: Sci Rep. 2023 Nov 14;13:19862. doi: 10.1038/s41598-023-46880-9 (PMC10646083; doi:10.1038/s41598-023-46880-9)
Supplement: Supplementary file 1 — Supplementary Information 1. [file 41598_2023_46880_MOESM1_ESM.pptx]

## Slide 1
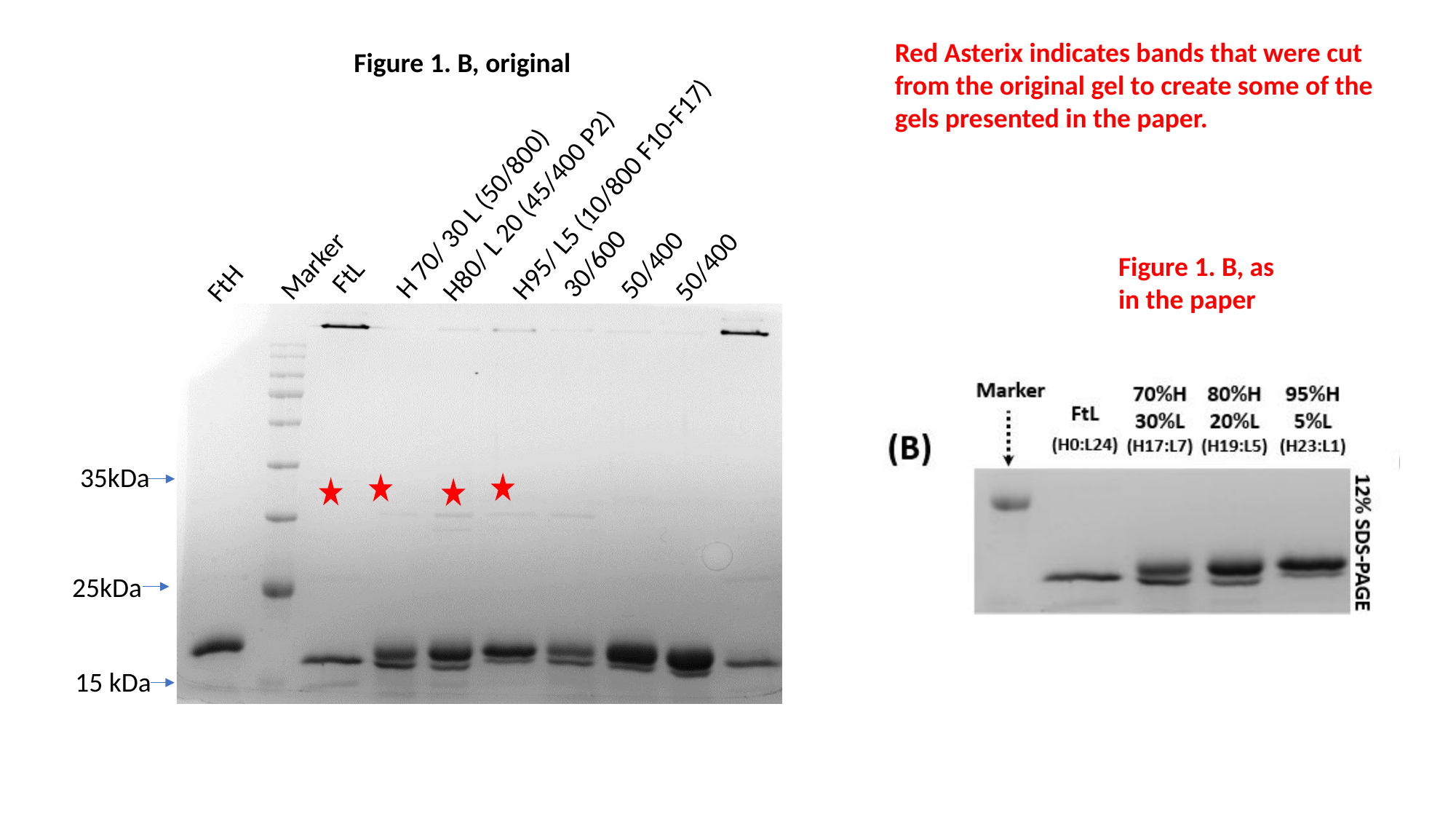

Red Asterix indicates bands that were cut from the original gel to create some of the gels presented in the paper.
Figure 1. B, original
H95/ L5 (10/800 F10-F17)
H80/ L 20 (45/400 P2)
H 70/ 30 L (50/800)
30/600
50/400
Marker
50/400
Figure 1. B, as in the paper
 FtL
FtH
35kDa
25kDa
15 kDa

## Slide 2
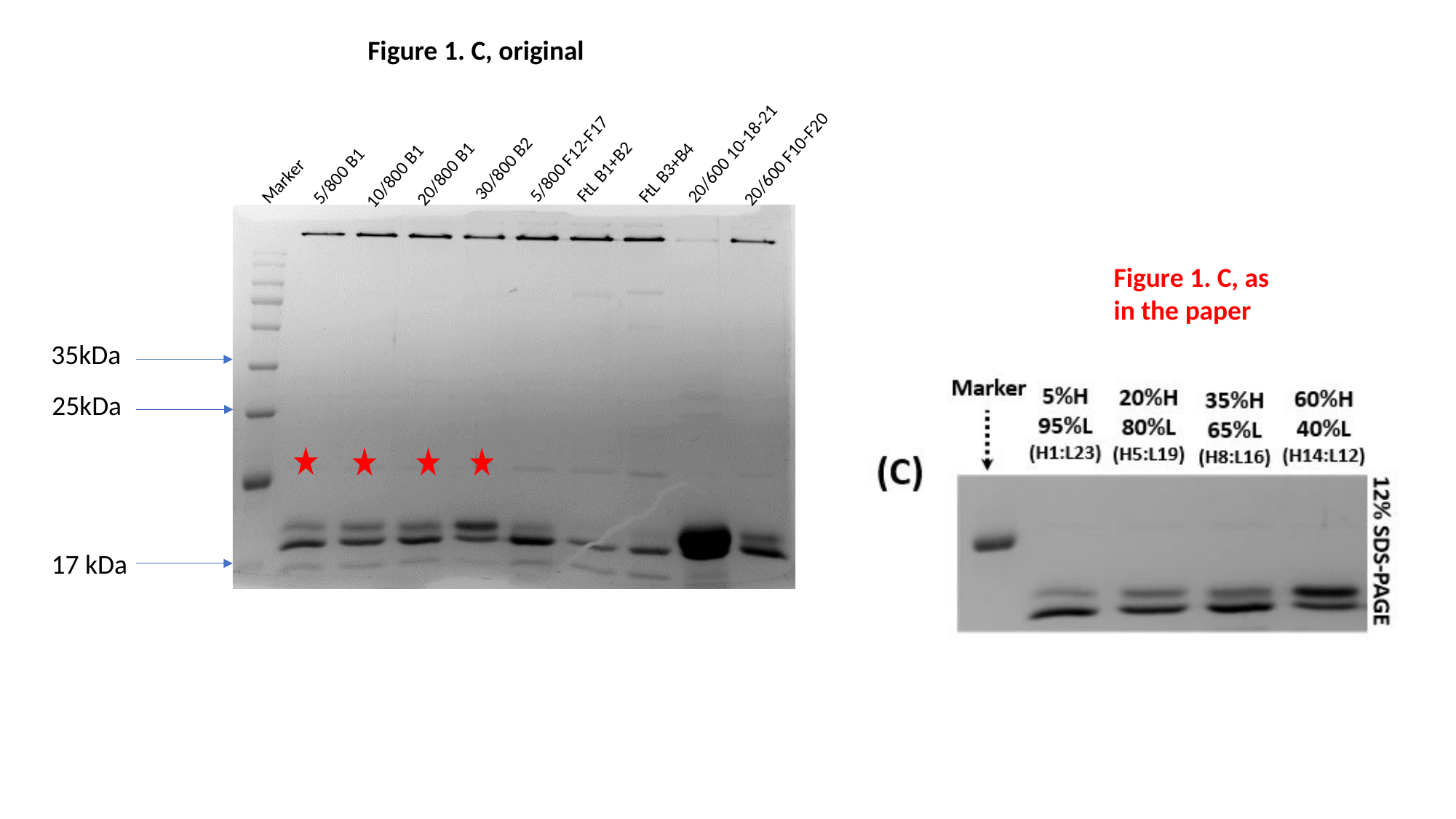

Figure 1. C, original
20/600 10-18-21
20/600 F10-F20
5/800 F12-F17
30/800 B2
FtL B1+B2
FtL B3+B4
20/800 B1
10/800 B1
5/800 B1
Marker
Figure 1. C, as in the paper
35kDa
25kDa
17 kDa

## Slide 3
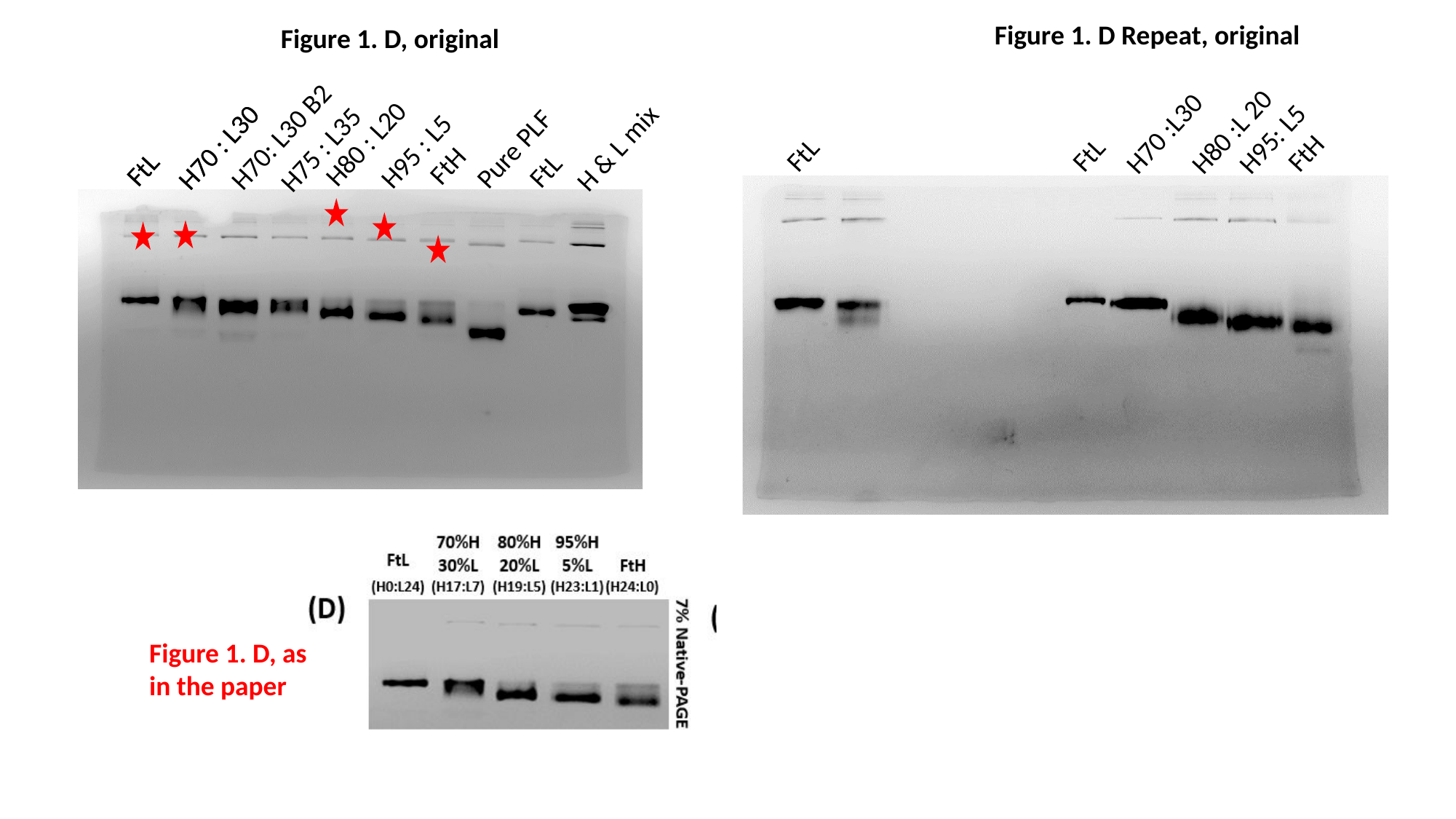

Figure 1. D Repeat, original
Figure 1. D, original
H80 :L 20
H70 :L30
H70: L30 B2
H95: L5
H80 : L20
H & L mix
Pure PLF
H95 : L5
H70 : L30
H70 : L30
H75 : L35
FtL
FtH
FtL
FtH
FtL
FtL
FtL
Figure 1. D, as in the paper

## Slide 4
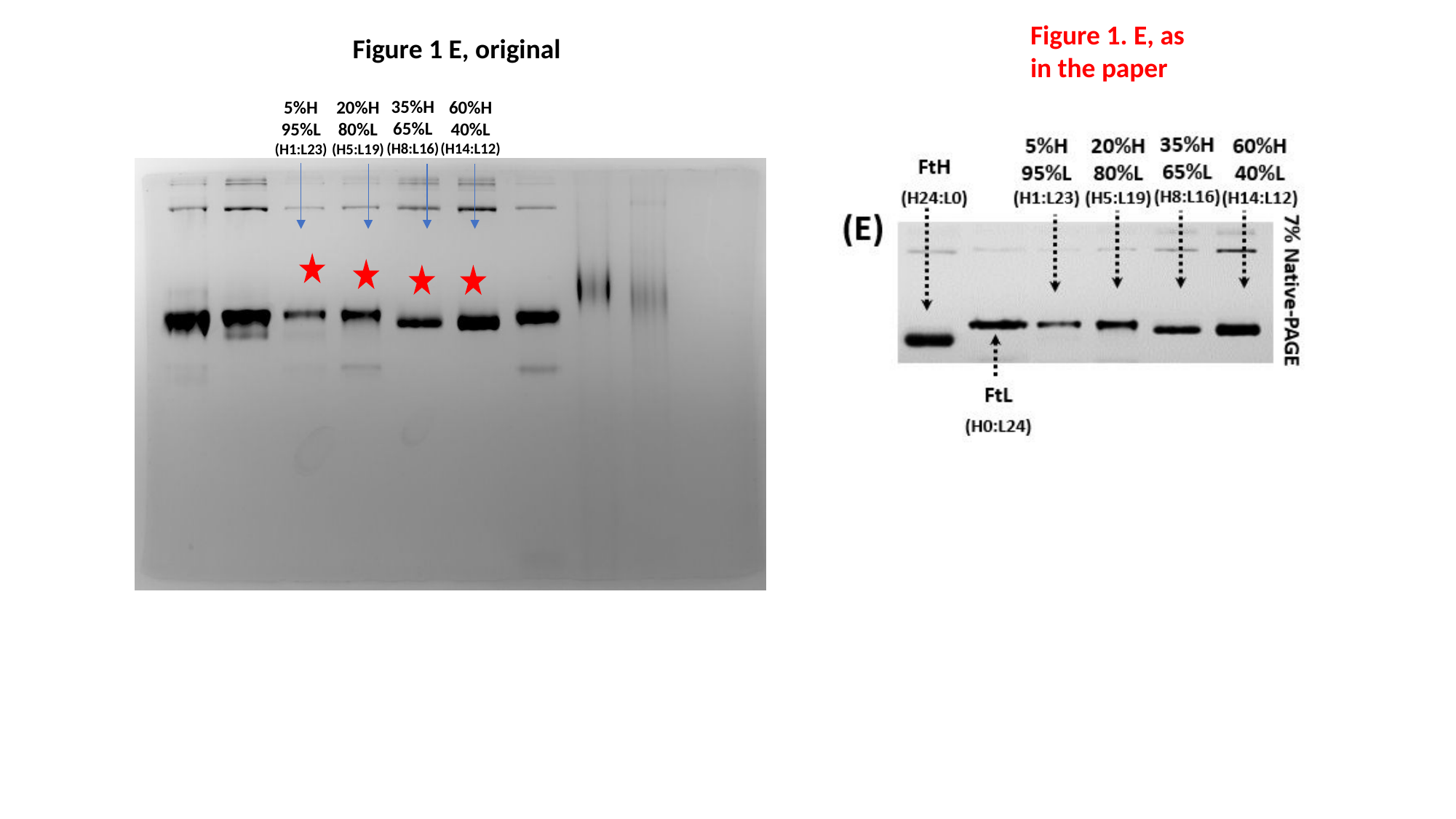

Figure 1. E, as in the paper
Figure 1 E, original
35%H
65%L
(H8:L16)
60%H
40%L
(H14:L12)
20%H
80%L
(H5:L19)
5%H
95%L
(H1:L23)

## Slide 5
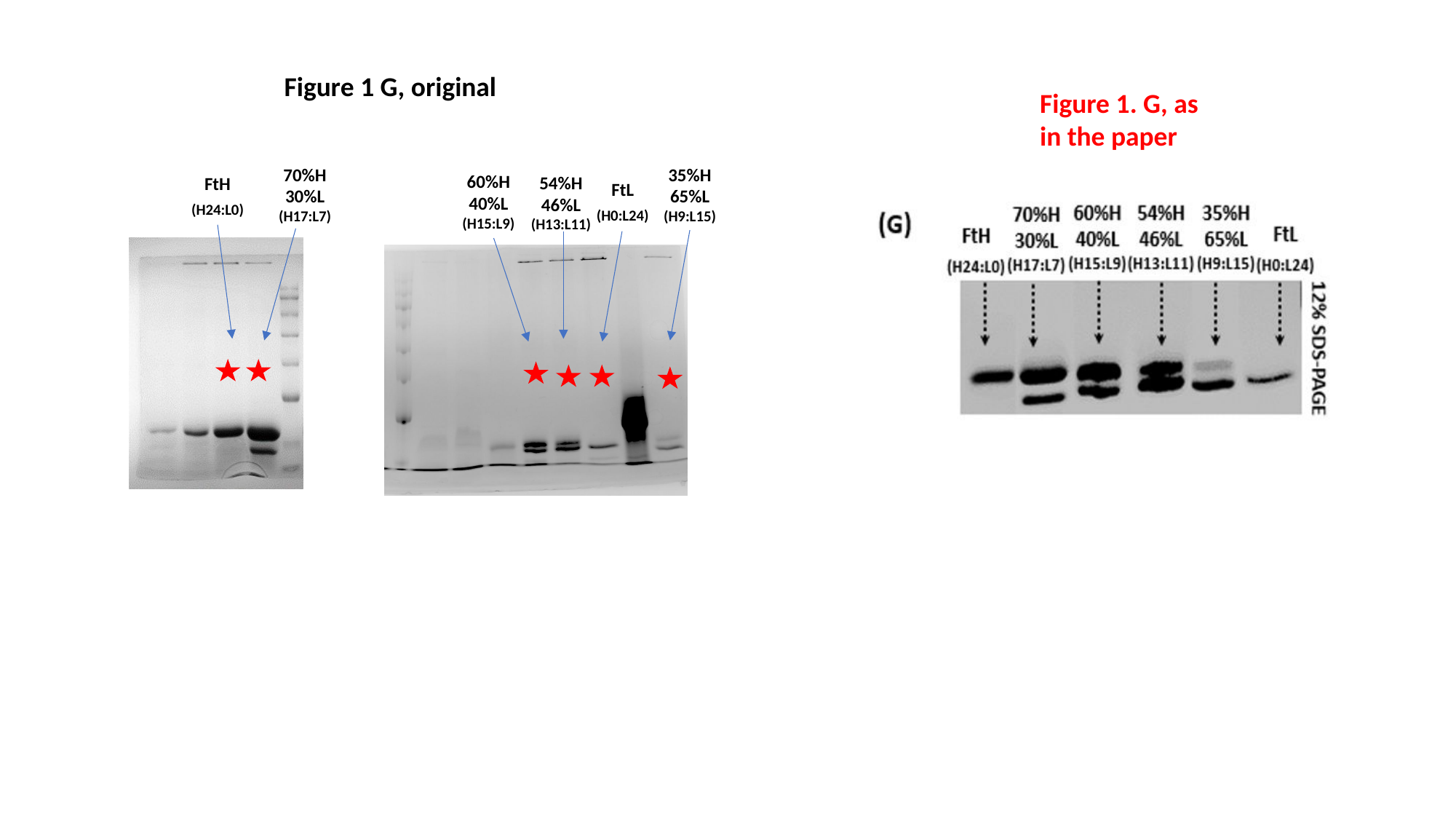

Figure 1 G, original
Figure 1. G, as in the paper
70%H
30%L
(H17:L7)
35%H
65%L
(H9:L15)
60%H
40%L
(H15:L9)
54%H
46%L
(H13:L11)
FtH
(H24:L0)
FtL
(H0:L24)

## Slide 6
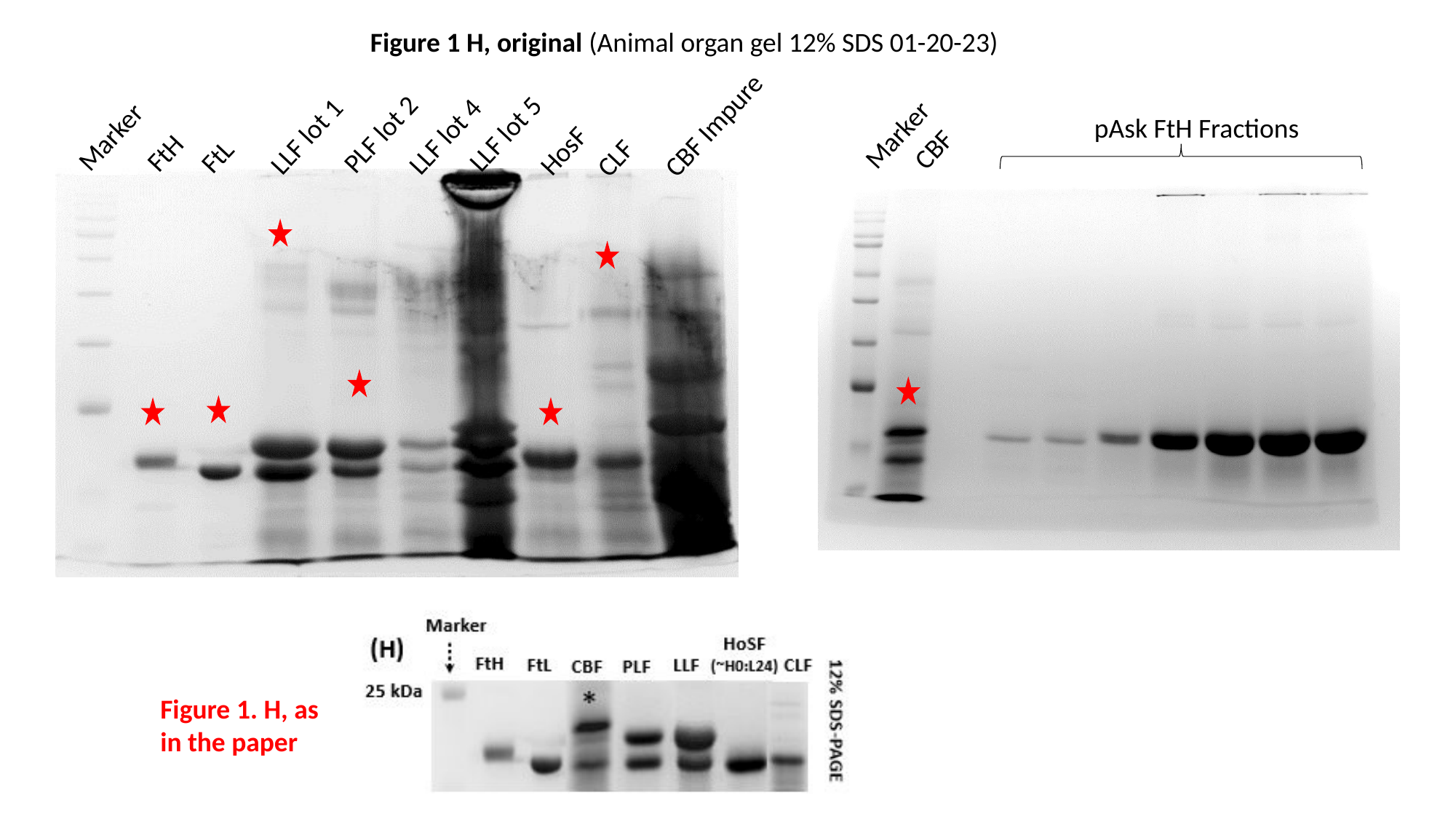

Figure 1 H, original (Animal organ gel 12% SDS 01-20-23)
CBF Impure
PLF lot 2
pAsk FtH Fractions
LLF lot 1
LLF lot 4
LLF lot 5
Marker
Marker
HosF
FtH
CBF
CLF
FtL
Figure 1. H, as in the paper

## Slide 7
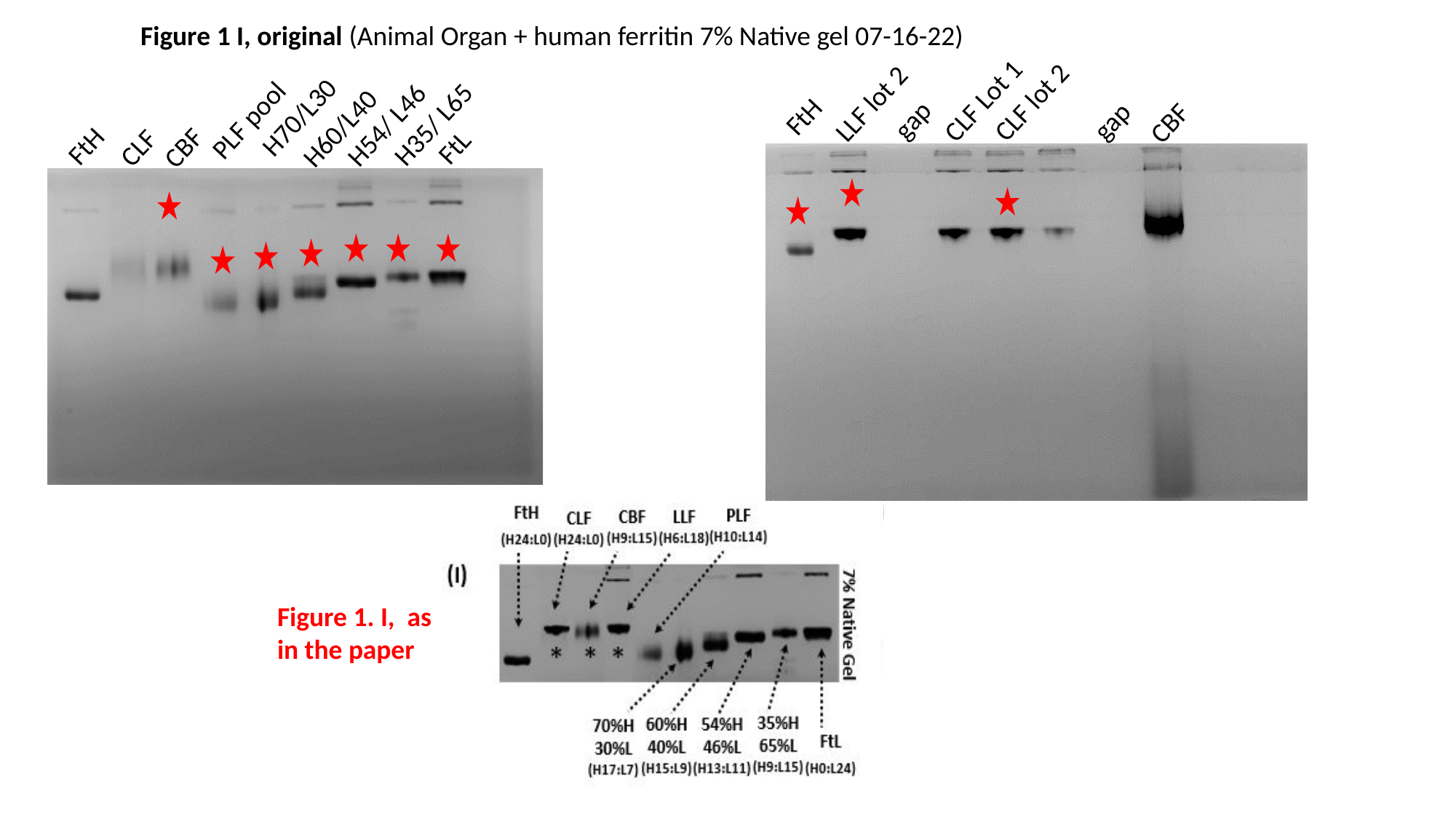

Figure 1 I, original (Animal Organ + human ferritin 7% Native gel 07-16-22)
CLF Lot 1
LLF lot 2
CLF lot 2
FtH
gap
CBF
gap
H70/L30
PLF pool
H35/ L65
H54/ L46
H60/L40
CLF
FtH
FtL
CBF
Figure 1. I, as in the paper

## Slide 8
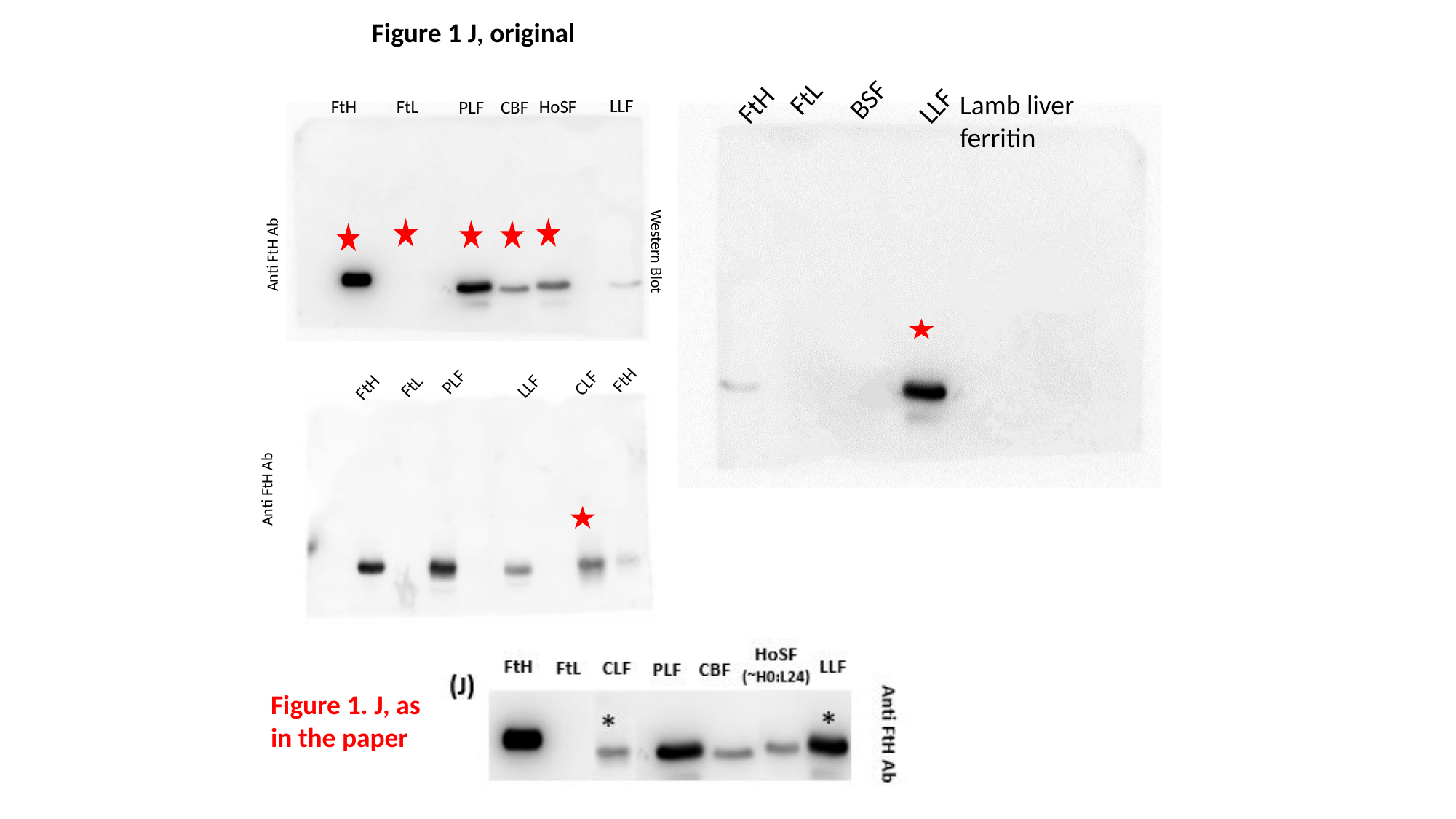

Figure 1 J, original
FtL
BSF
LLF
FtH
Lamb liver ferritin
LLF
FtH
FtL
HoSF
PLF
CBF
Anti FtH Ab
Western Blot
FtH
CLF
PLF
FtH
LLF
FtL
Anti FtH Ab
Figure 1. J, as
in the paper

## Slide 9
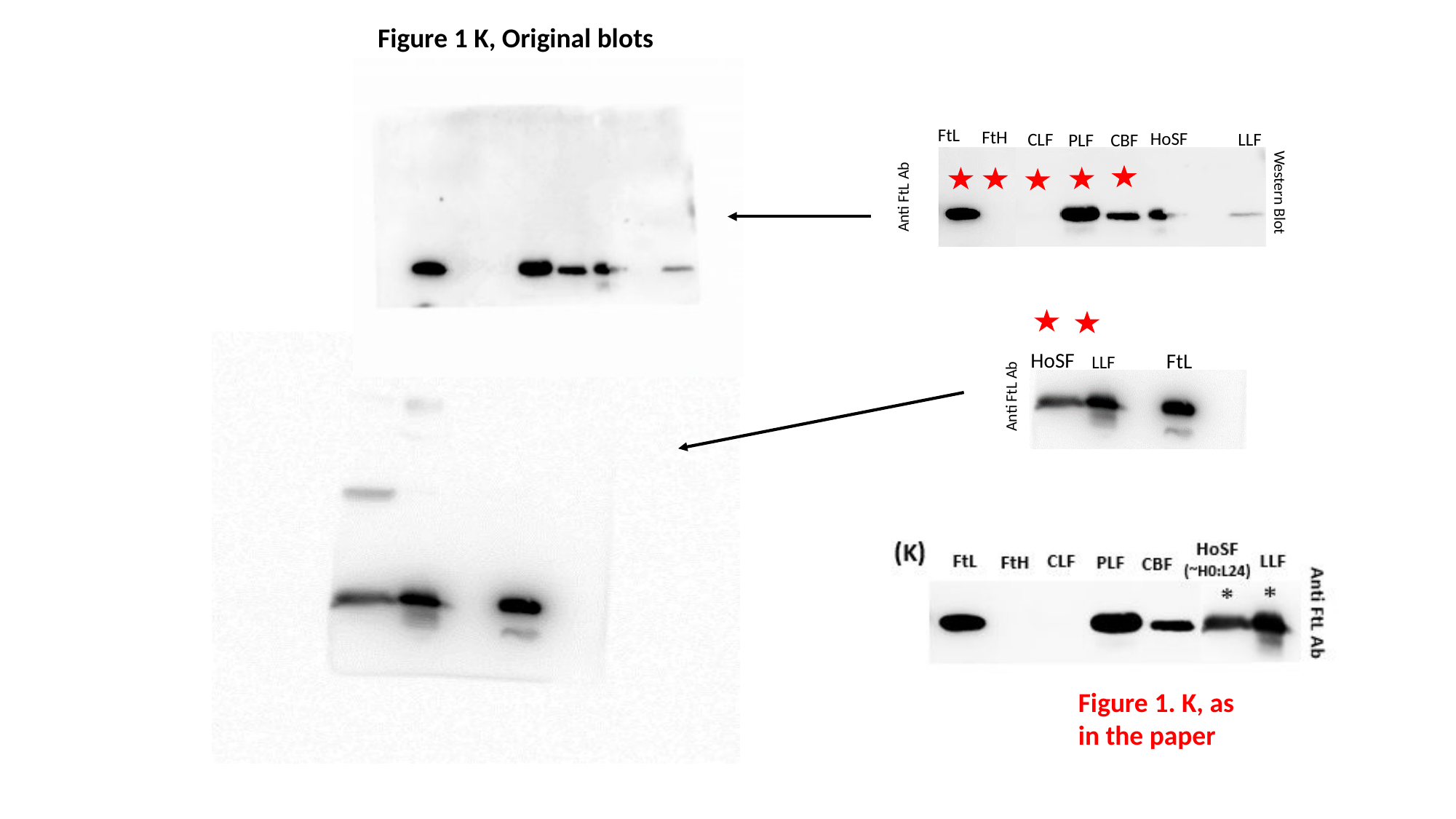

Figure 1 K, Original blots
FtL
FtH
HoSF
LLF
PLF
CBF
Anti FtL Ab
Western Blot
CLF
HoSF
FtL
LLF
Anti FtL Ab
Figure 1. K, as
in the paper

## Slide 10
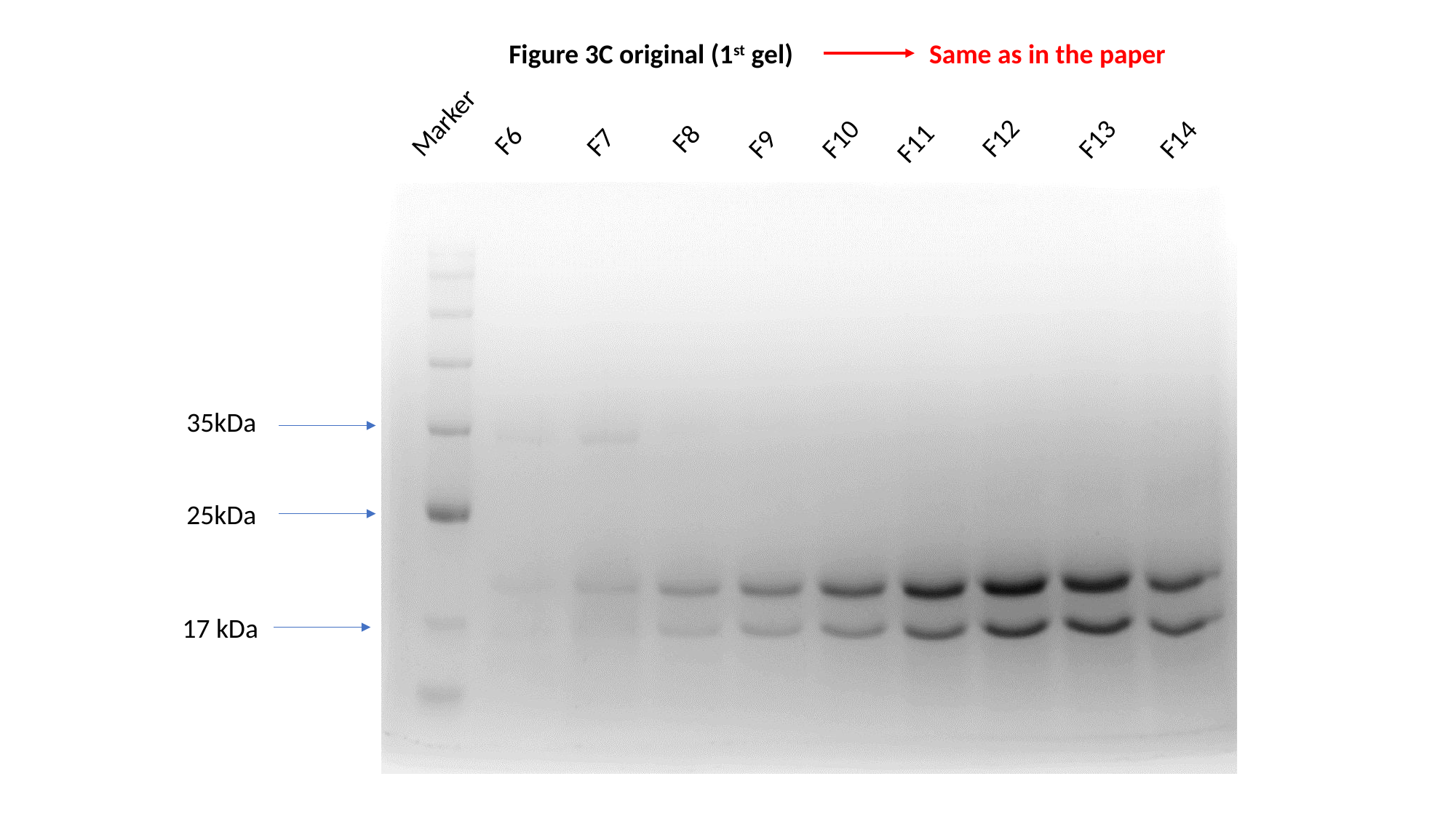

Figure 3C original (1st gel) Same as in the paper
F8
F6
F7
Marker
F12
F9
F10
F13
F14
F11
35kDa
25kDa
17 kDa

## Slide 11
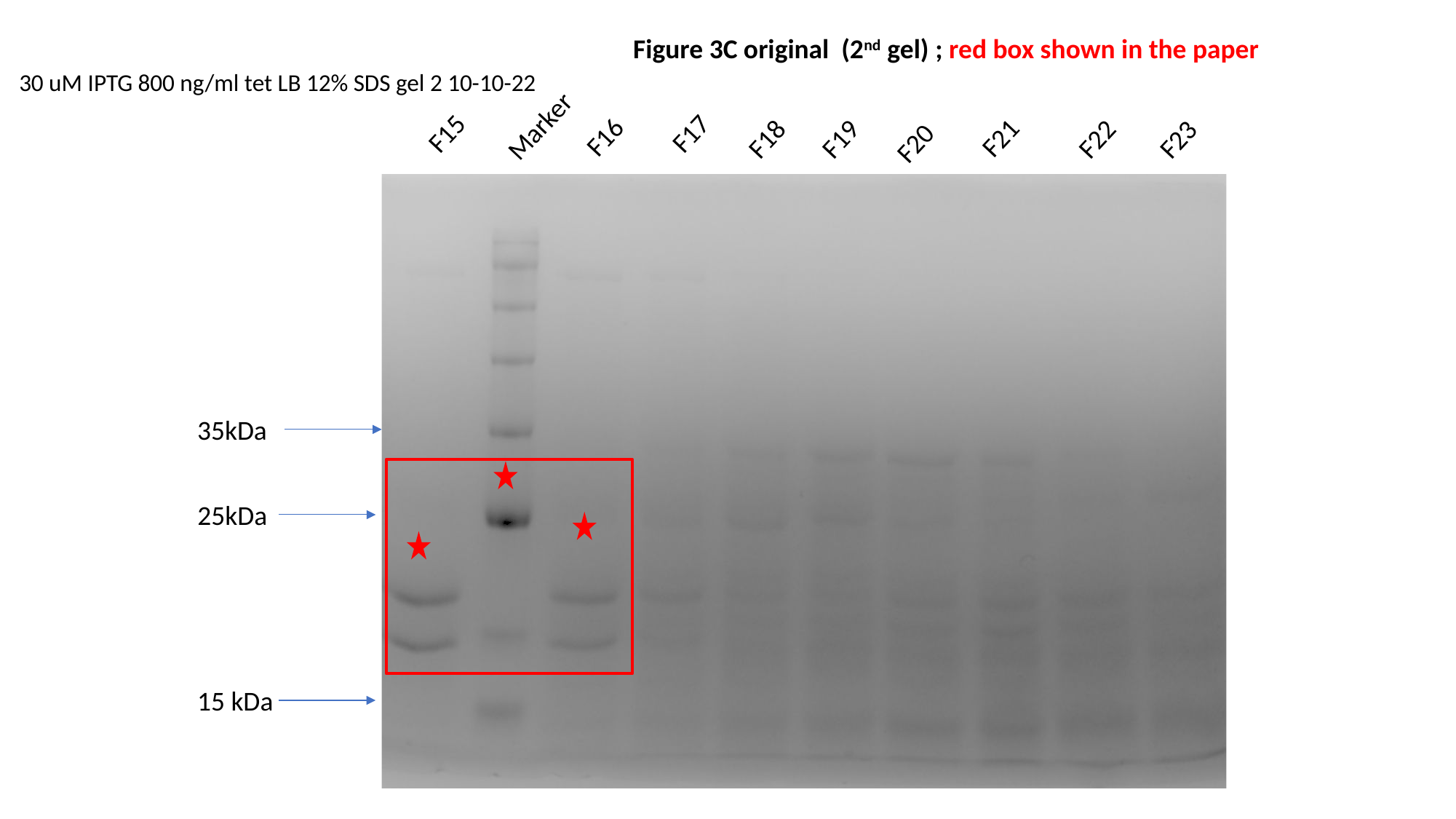

Figure 3C original (2nd gel) ; red box shown in the paper
30 uM IPTG 800 ng/ml tet LB 12% SDS gel 2 10-10-22
F17
F15
F16
F21
F18
F19
F22
F23
Marker
F20
35kDa
25kDa
15 kDa

## Slide 12
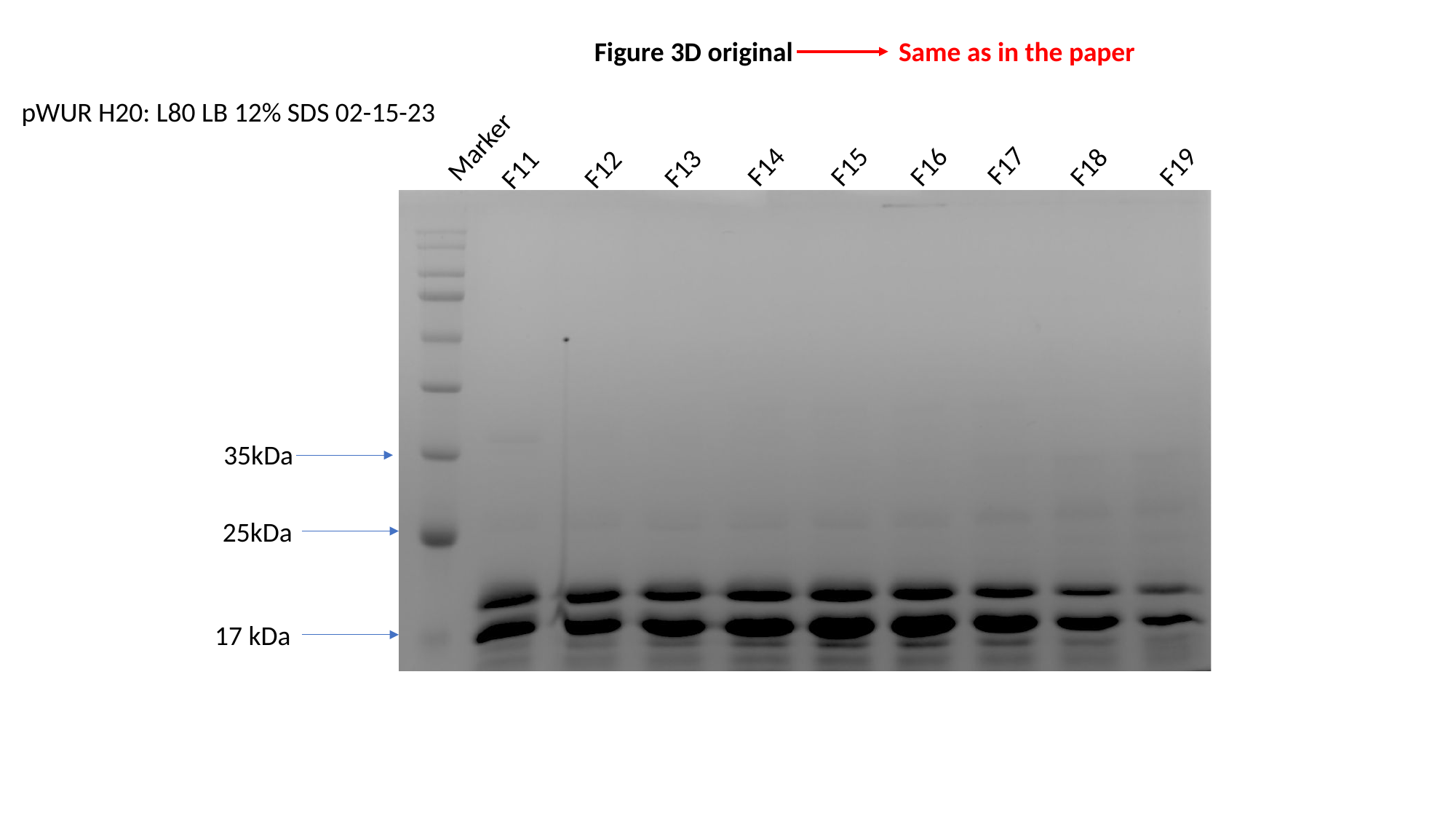

Figure 3D original Same as in the paper
pWUR H20: L80 LB 12% SDS 02-15-23
Marker
F14
F17
F19
F15
F16
F18
F13
F12
F11
35kDa
25kDa
17 kDa

## Slide 13
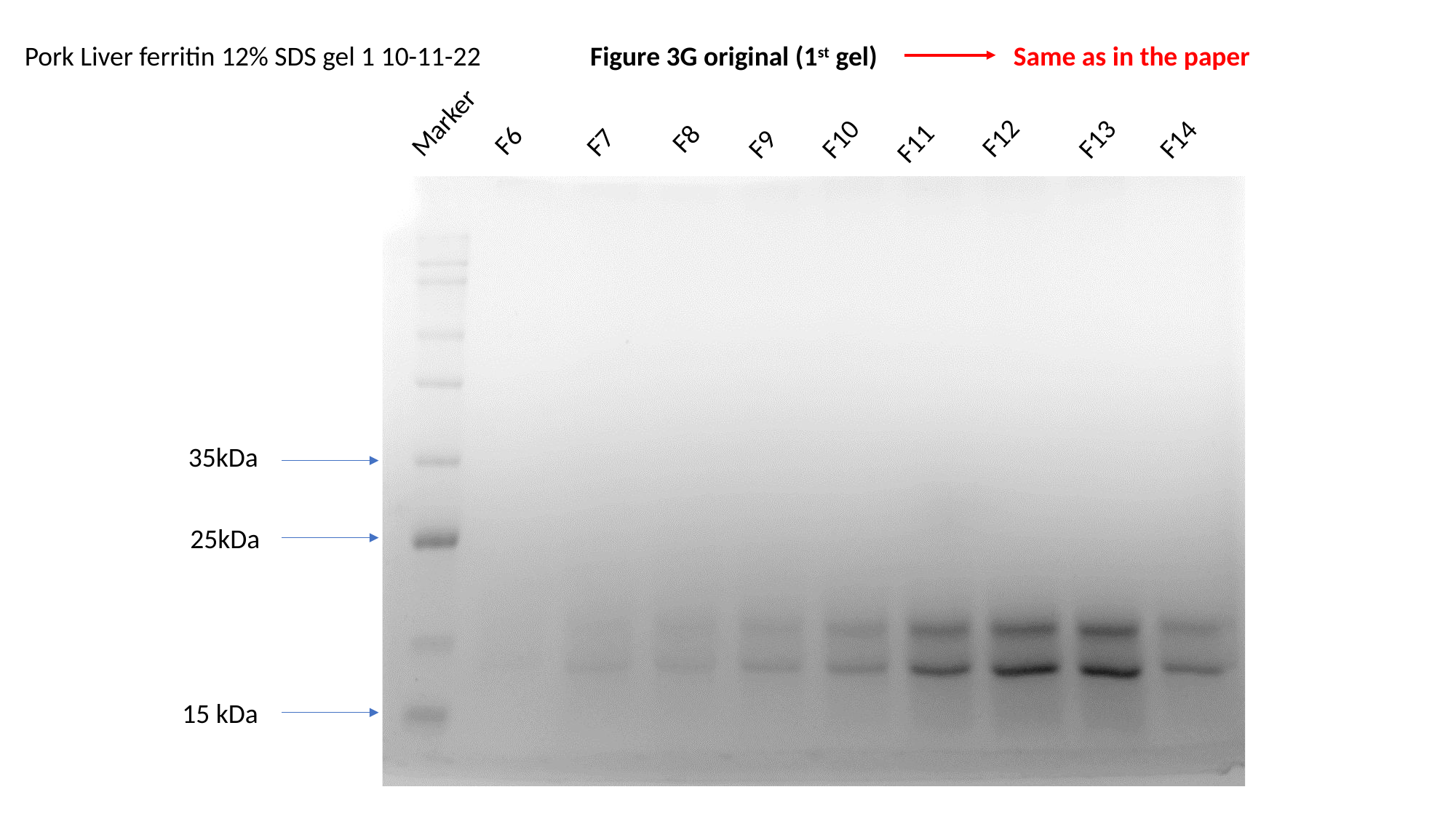

Figure 3G original (1st gel) Same as in the paper
Pork Liver ferritin 12% SDS gel 1 10-11-22
F8
F6
F7
Marker
F12
F9
F10
F13
F14
F11
35kDa
25kDa
15 kDa

## Slide 14
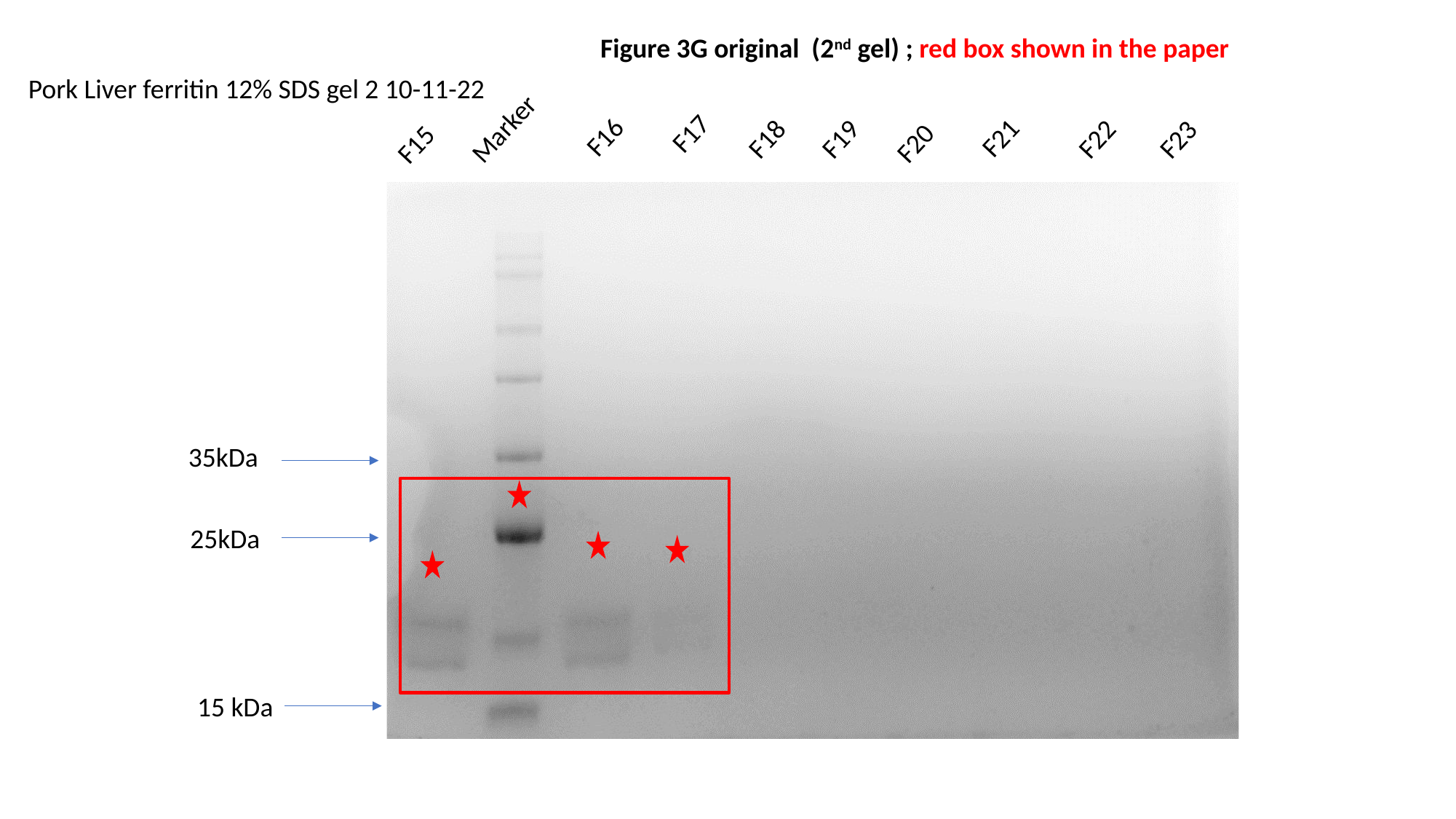

Figure 3G original (2nd gel) ; red box shown in the paper
Pork Liver ferritin 12% SDS gel 2 10-11-22
F17
F16
F21
F18
F19
F22
F23
F20
Marker
F15
35kDa
25kDa
15 kDa

## Slide 15
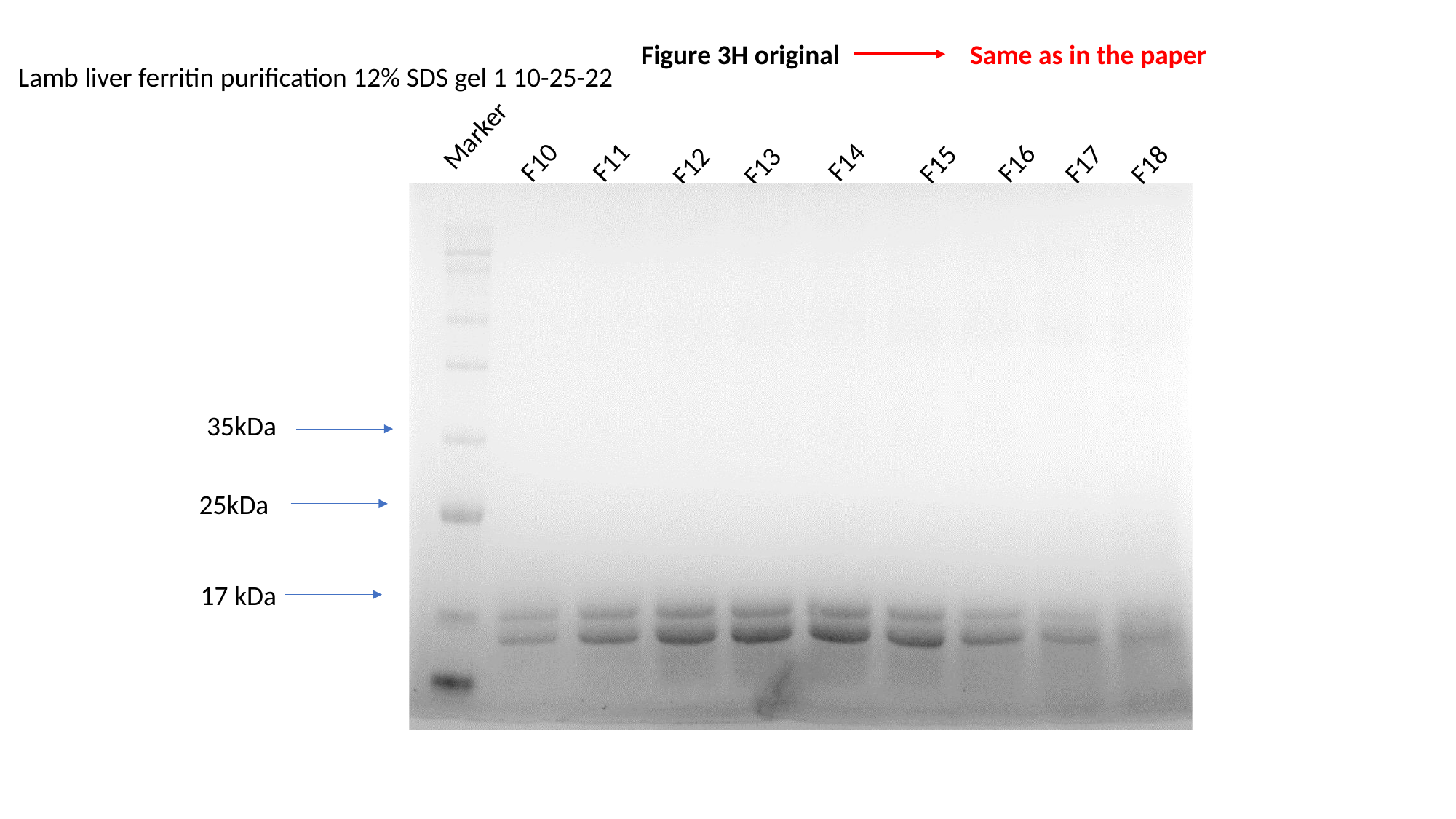

Figure 3H original Same as in the paper
Lamb liver ferritin purification 12% SDS gel 1 10-25-22
Marker
F11
F14
F10
F16
F12
F15
F17
F18
F13
35kDa
25kDa
17 kDa

## Slide 16
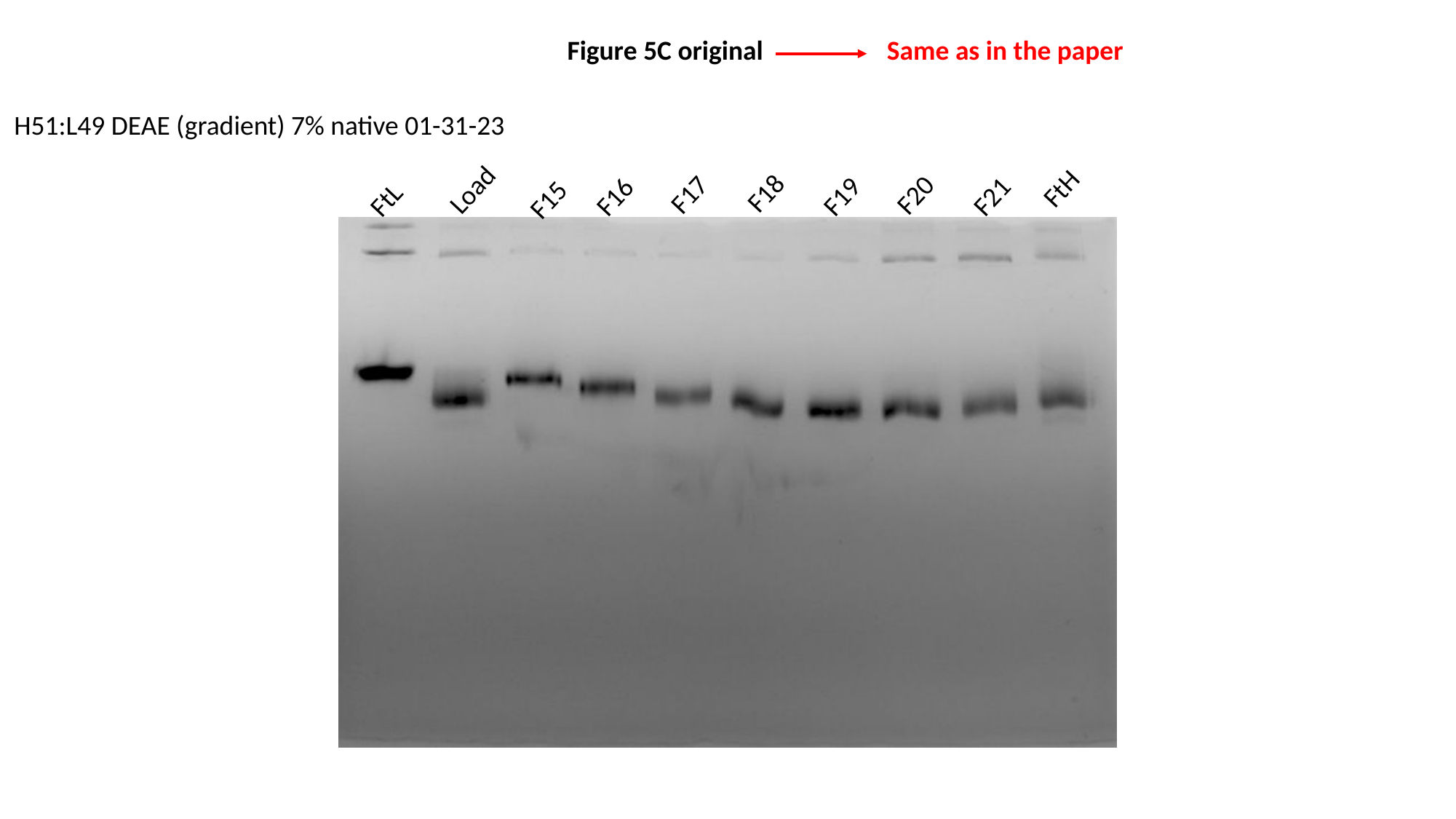

Figure 5C original Same as in the paper
H51:L49 DEAE (gradient) 7% native 01-31-23
FtL
Load
FtH
F21
F17
F18
F19
F20
F16
F15

## Slide 17
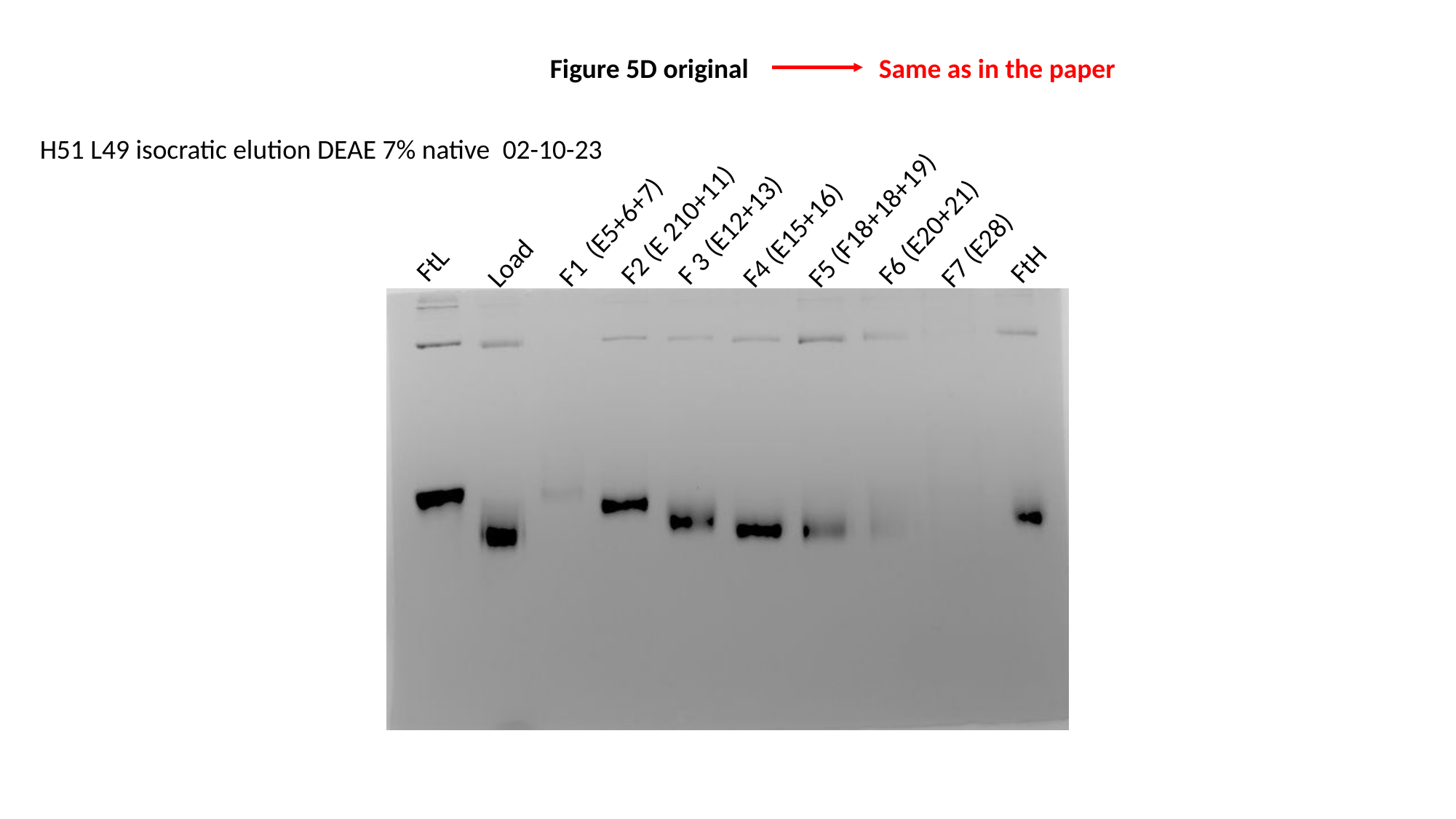

Figure 5D original Same as in the paper
H51 L49 isocratic elution DEAE 7% native  02-10-23
F5 (F18+18+19)
F1 (E5+6+7)
F2 (E 210+11)
F 3 (E12+13)
F4 (E15+16)
F6 (E20+21)
F7 (E28)
Load
FtL
FtH

## Slide 18
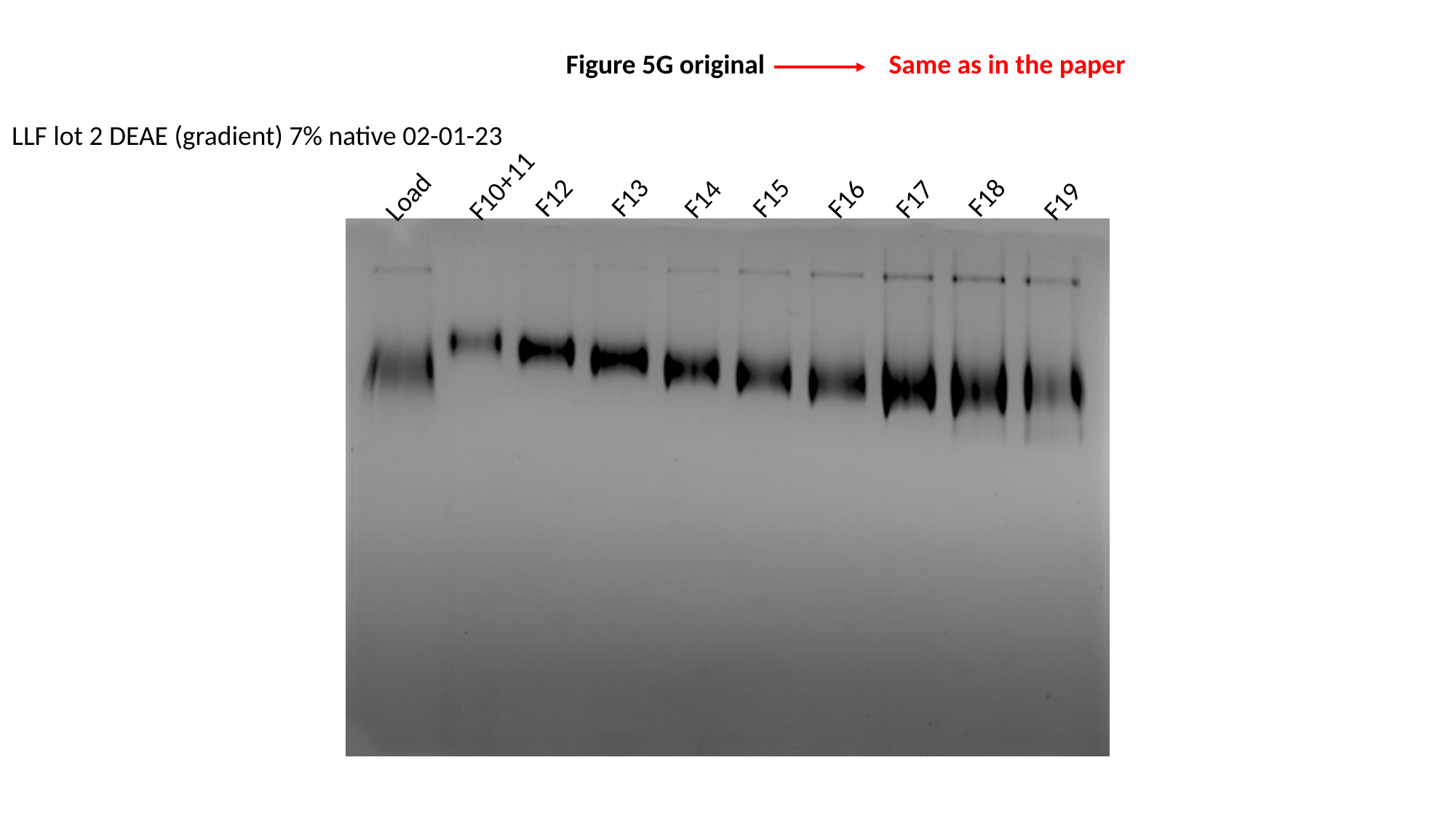

Figure 5G original Same as in the paper
LLF lot 2 DEAE (gradient) 7% native 02-01-23
Load
F10+11
F17
F14
F15
F18
F12
F13
F16
F19

## Slide 19
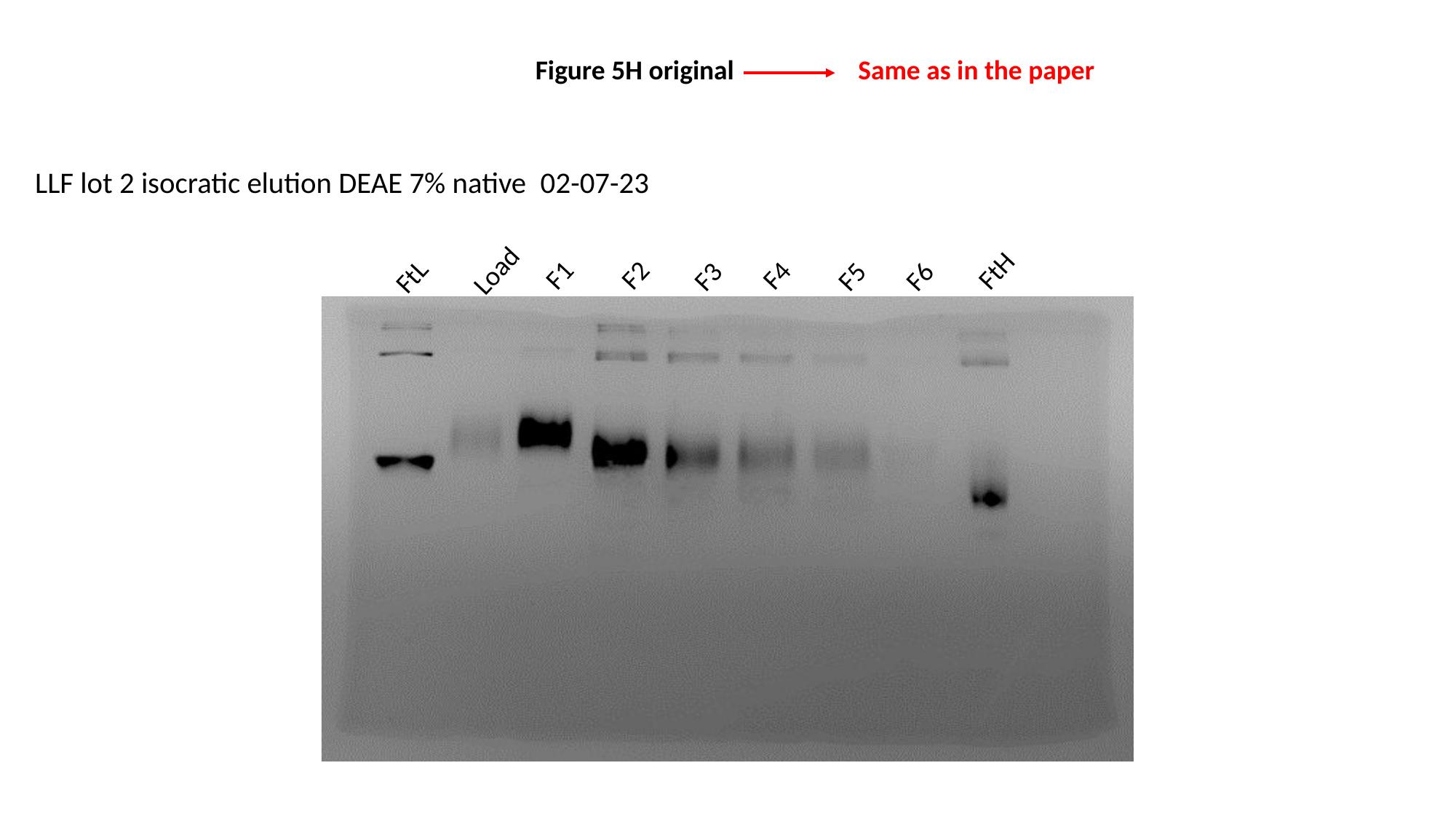

Figure 5H original Same as in the paper
LLF lot 2 isocratic elution DEAE 7% native  02-07-23
FtH
Load
F6
F3
F4
F1
F2
F5
FtL

## Slide 20
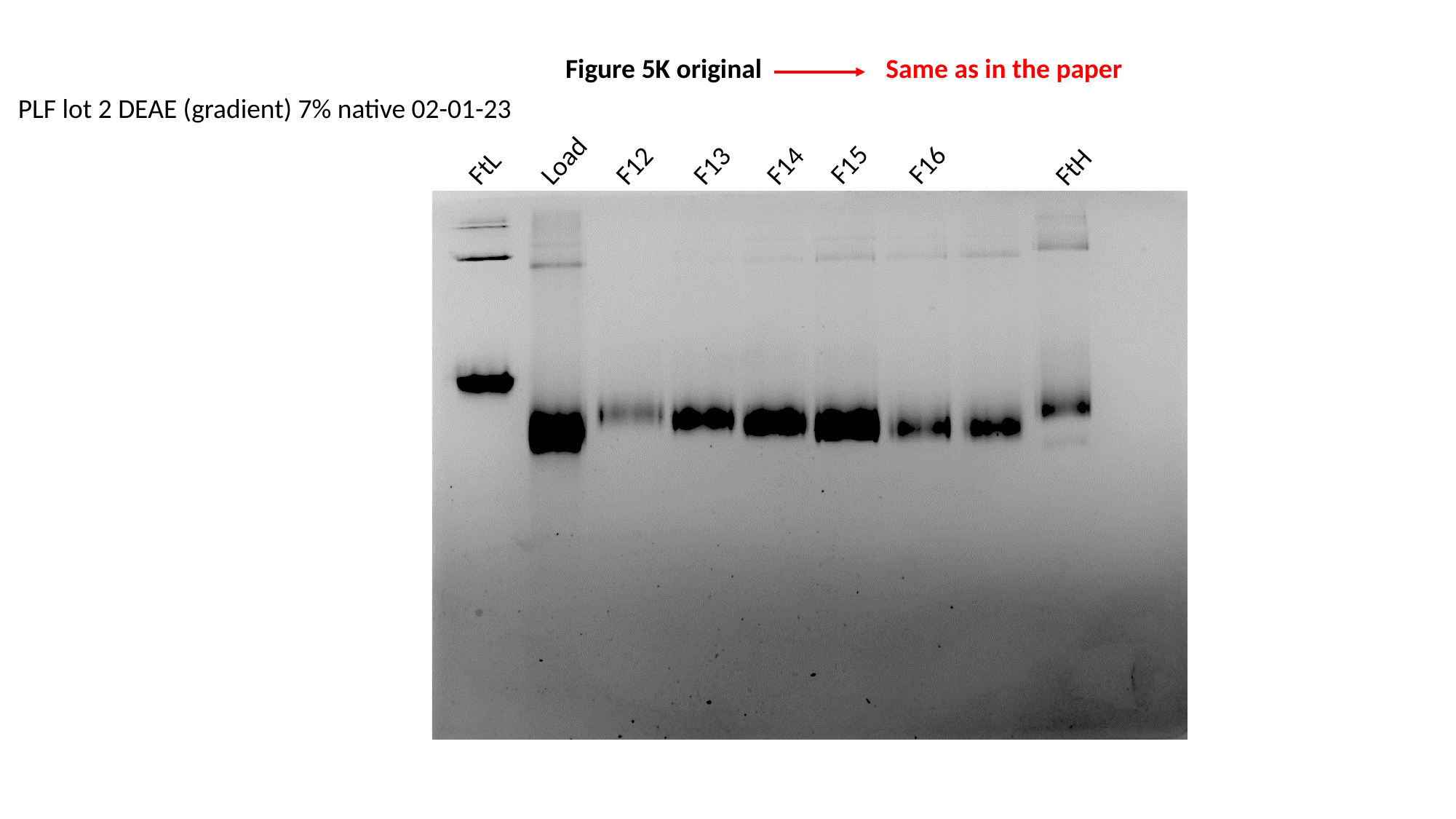

Figure 5K original Same as in the paper
PLF lot 2 DEAE (gradient) 7% native 02-01-23
Load
FtL
F13
FtH
F16
F12
F15
F14

## Slide 21
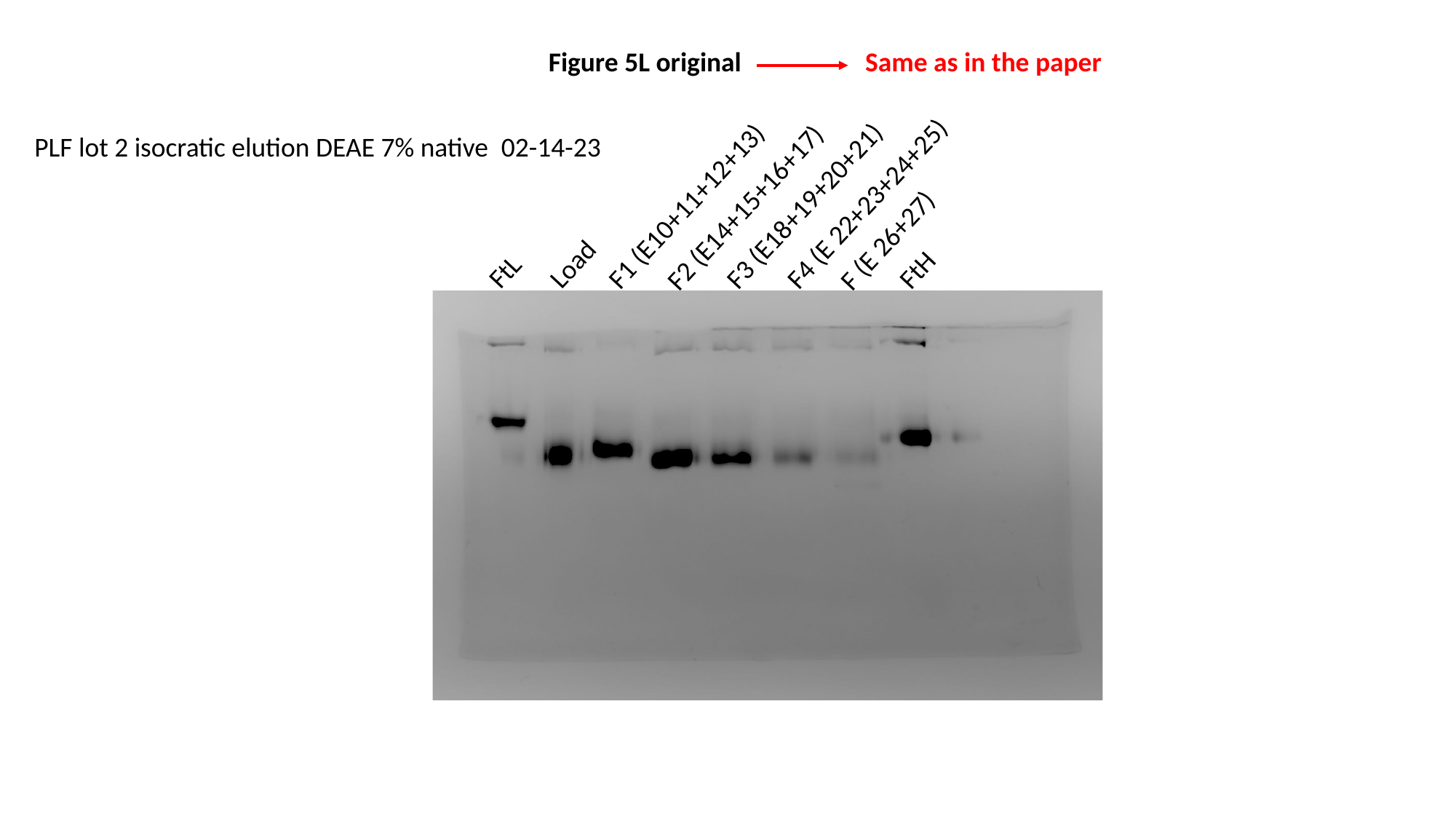

Figure 5L original Same as in the paper
PLF lot 2 isocratic elution DEAE 7% native  02-14-23
F1 (E10+11+12+13)
F4 (E 22+23+24+25)
F3 (E18+19+20+21)
F2 (E14+15+16+17)
F (E 26+27)
FtH
Load
FtL

## Slide 22
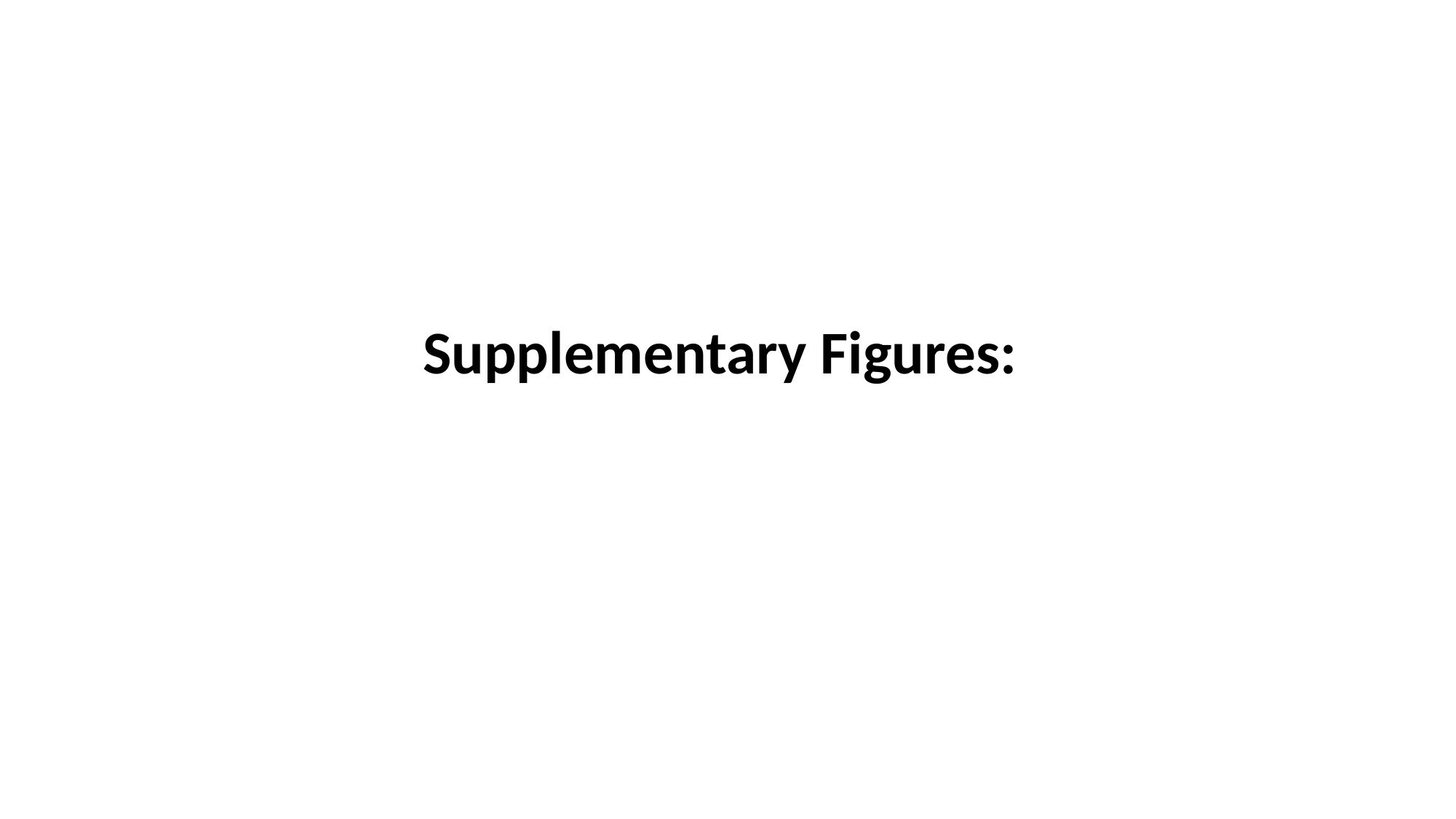

Supplementary Figures:

## Slide 23
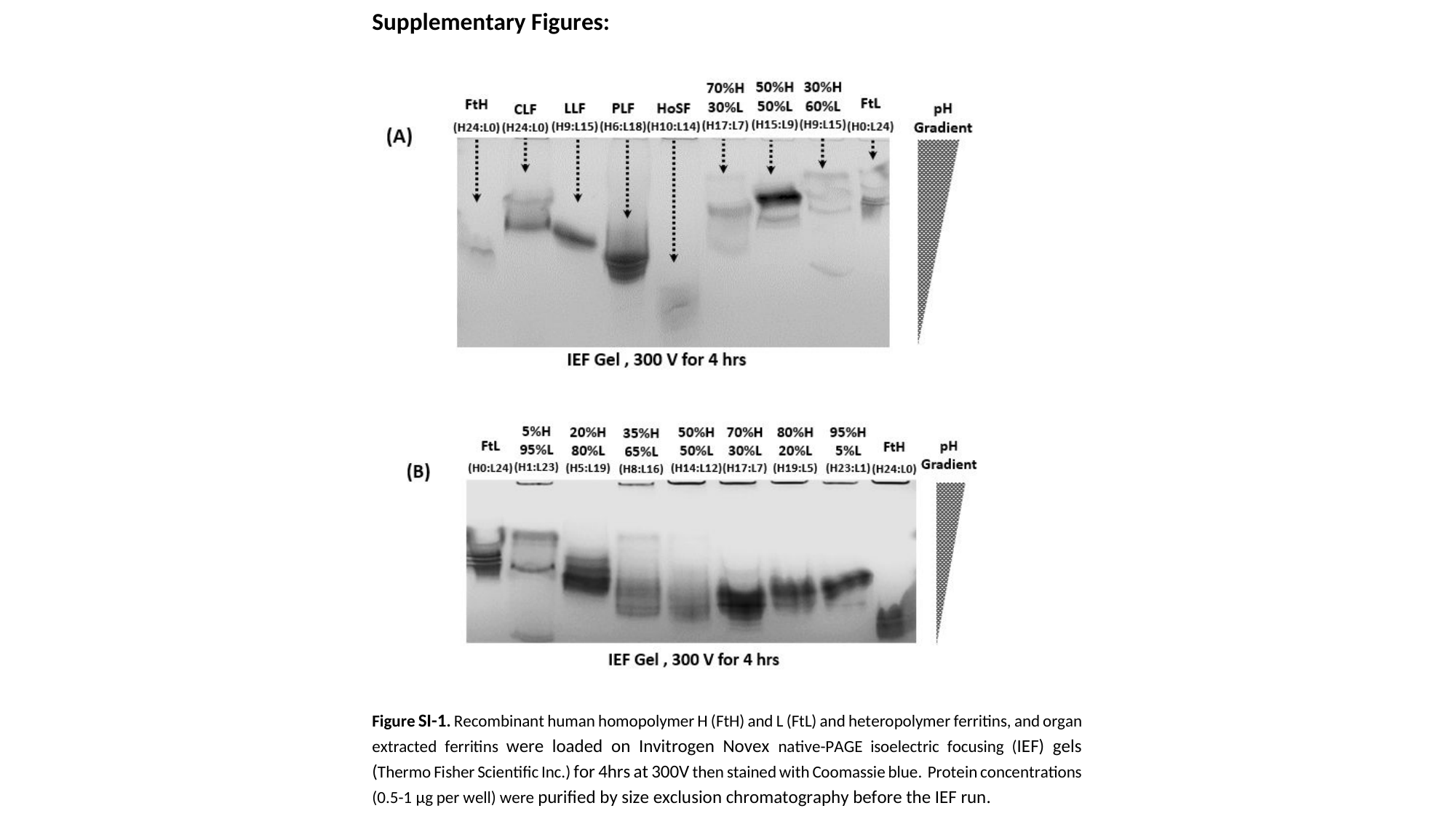

## Slide 24
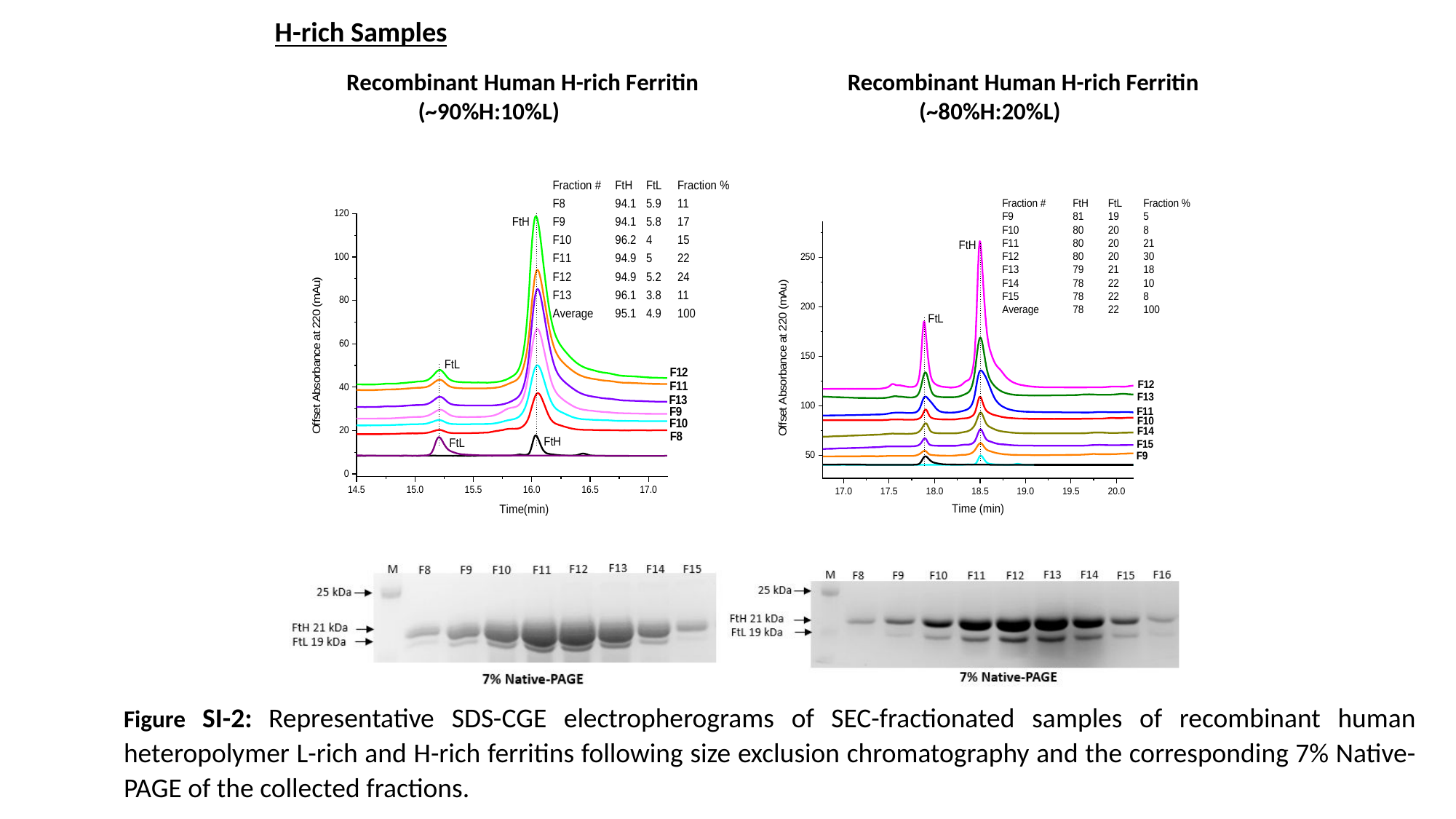

Figure SI-2: Representative SDS-CGE electropherograms of SEC-fractionated samples of recombinant human heteropolymer L-rich and H-rich ferritins following size exclusion chromatography and the corresponding 7% Native-PAGE of the collected fractions.

## Slide 25
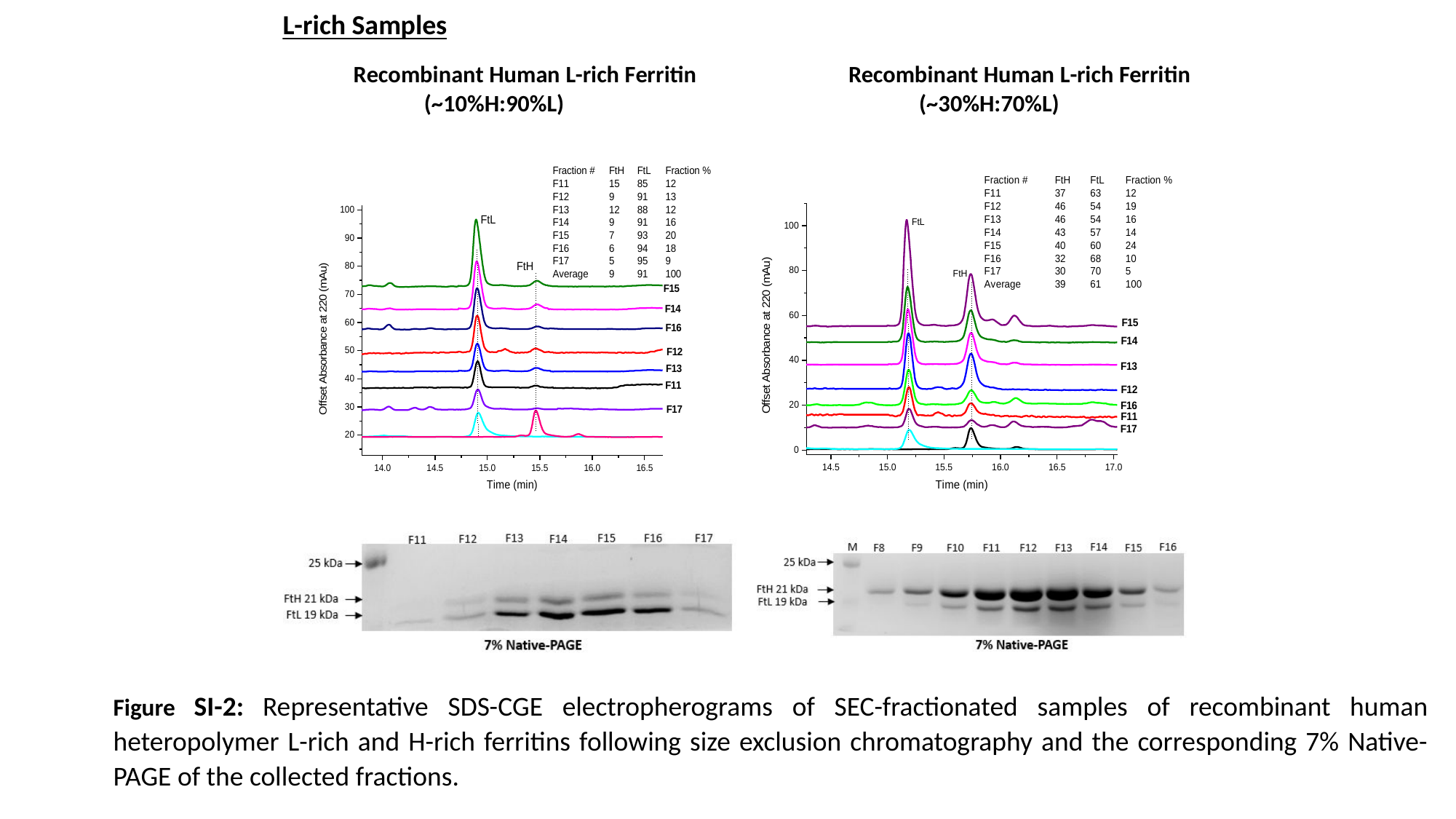

Figure SI-2: Representative SDS-CGE electropherograms of SEC-fractionated samples of recombinant human heteropolymer L-rich and H-rich ferritins following size exclusion chromatography and the corresponding 7% Native-PAGE of the collected fractions.

## Slide 26
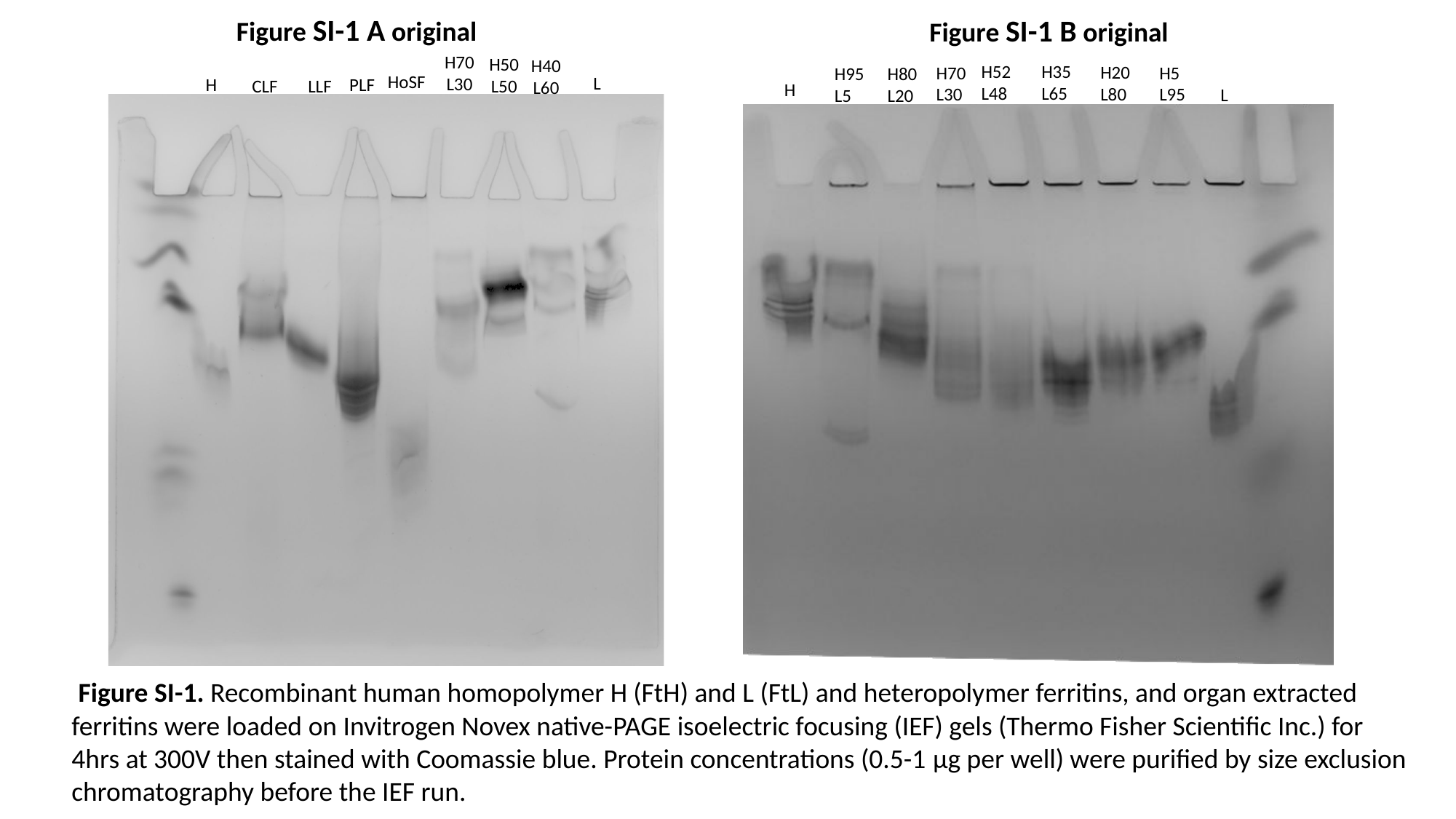

Figure SI-1 A original
Figure SI-1 B original
H70
L30
H50
L50
H40
L60
HoSF
L
H
PLF
LLF
CLF
H35
L65
H52
L48
H20
L80
H70
L30
H5
L95
H95
L5
H80
L20
H
L
 Figure SI-1. Recombinant human homopolymer H (FtH) and L (FtL) and heteropolymer ferritins, and organ extracted ferritins were loaded on Invitrogen Novex native-PAGE isoelectric focusing (IEF) gels (Thermo Fisher Scientific Inc.) for 4hrs at 300V then stained with Coomassie blue. Protein concentrations (0.5-1 μg per well) were purified by size exclusion chromatography before the IEF run.

## Slide 27
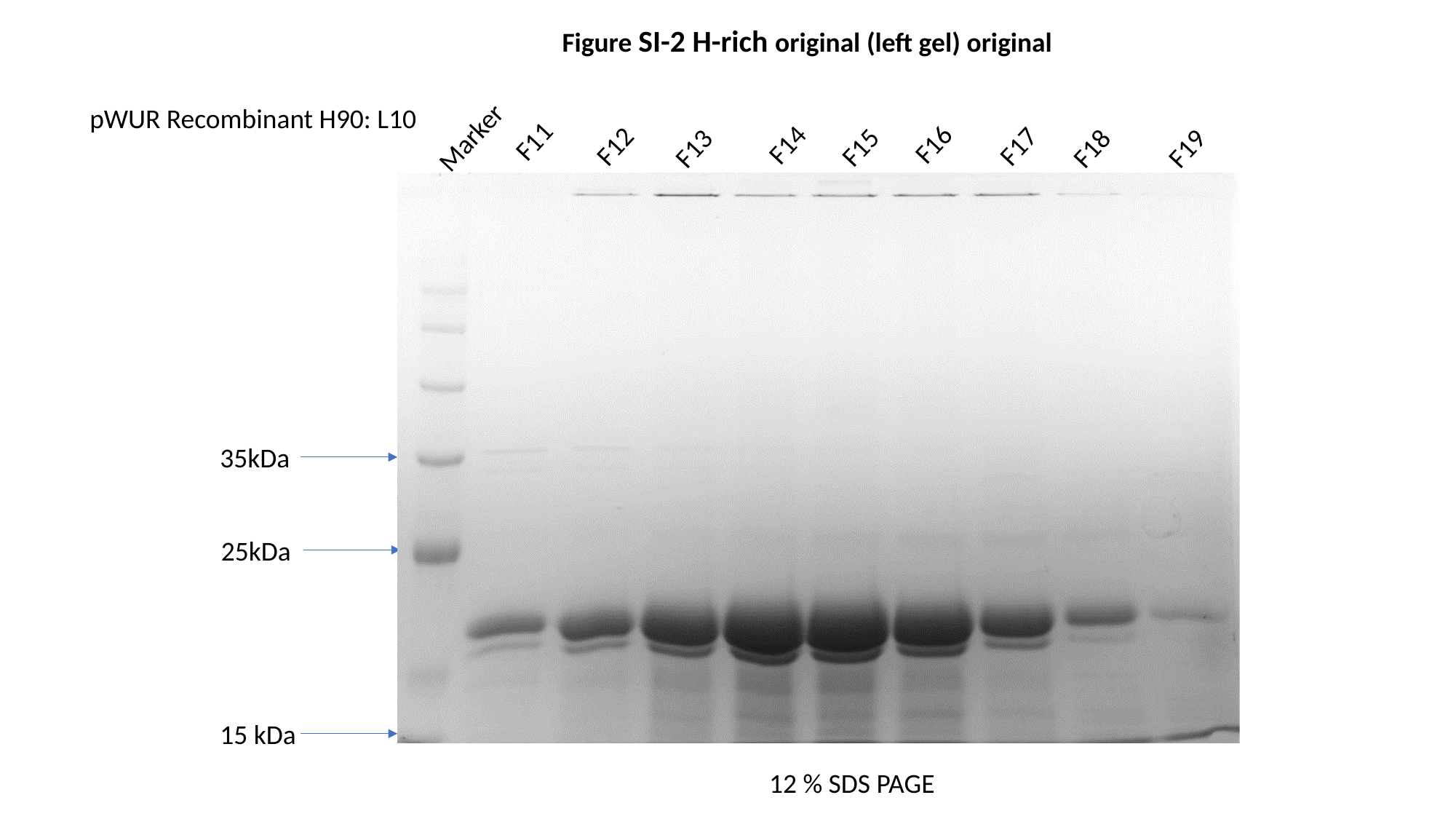

Figure SI-2 H-rich original (left gel) original
pWUR Recombinant H90: L10
F11
Marker
F16
F14
F12
F17
F15
F19
F13
F18
35kDa
25kDa
15 kDa
12 % SDS PAGE

## Slide 28
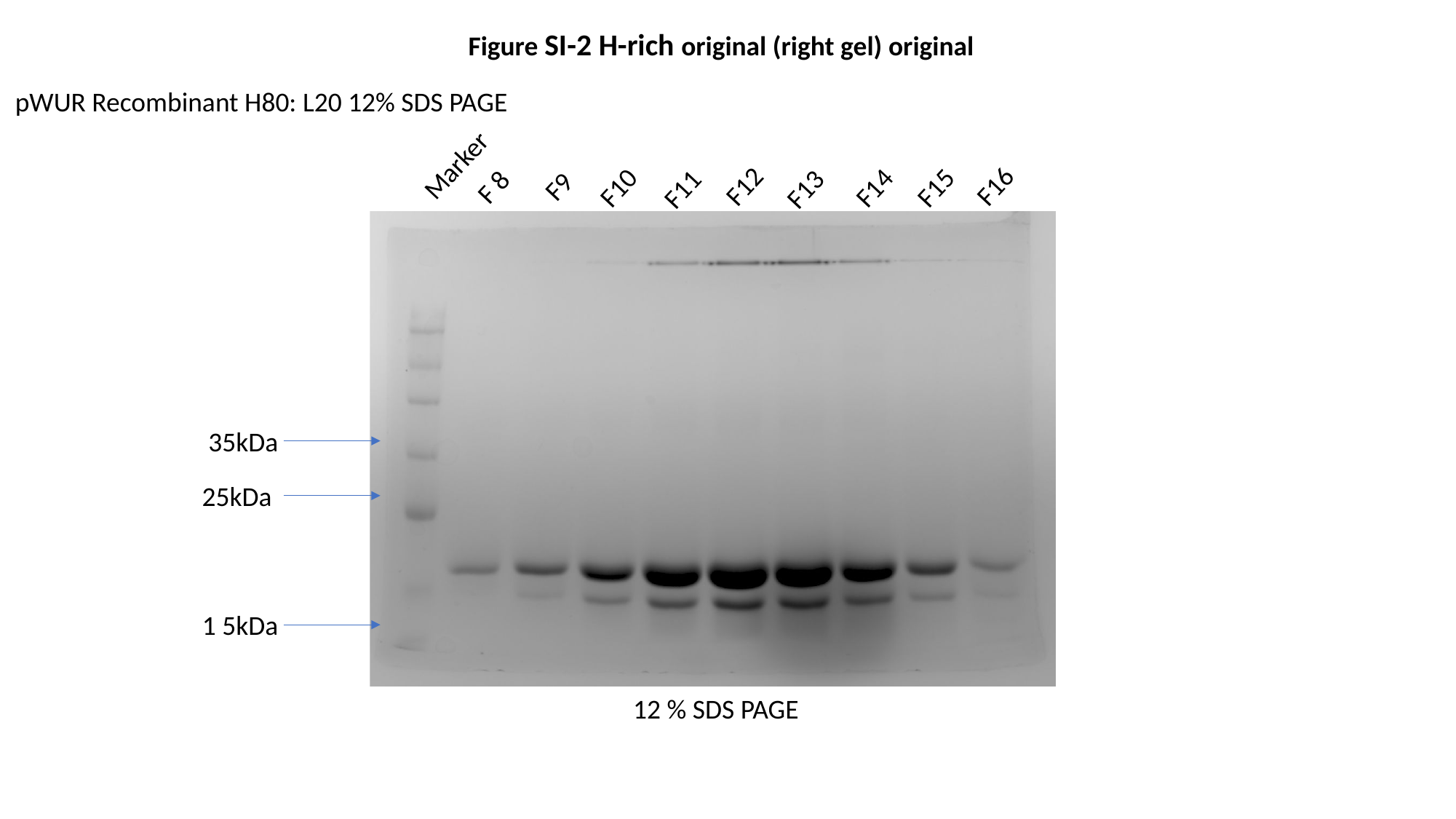

Figure SI-2 H-rich original (right gel) original
pWUR Recombinant H80: L20 12% SDS PAGE
Marker
F 8
F14
F16
F12
F9
F15
F10
F11
F13
35kDa
25kDa
1 5kDa
12 % SDS PAGE

## Slide 29
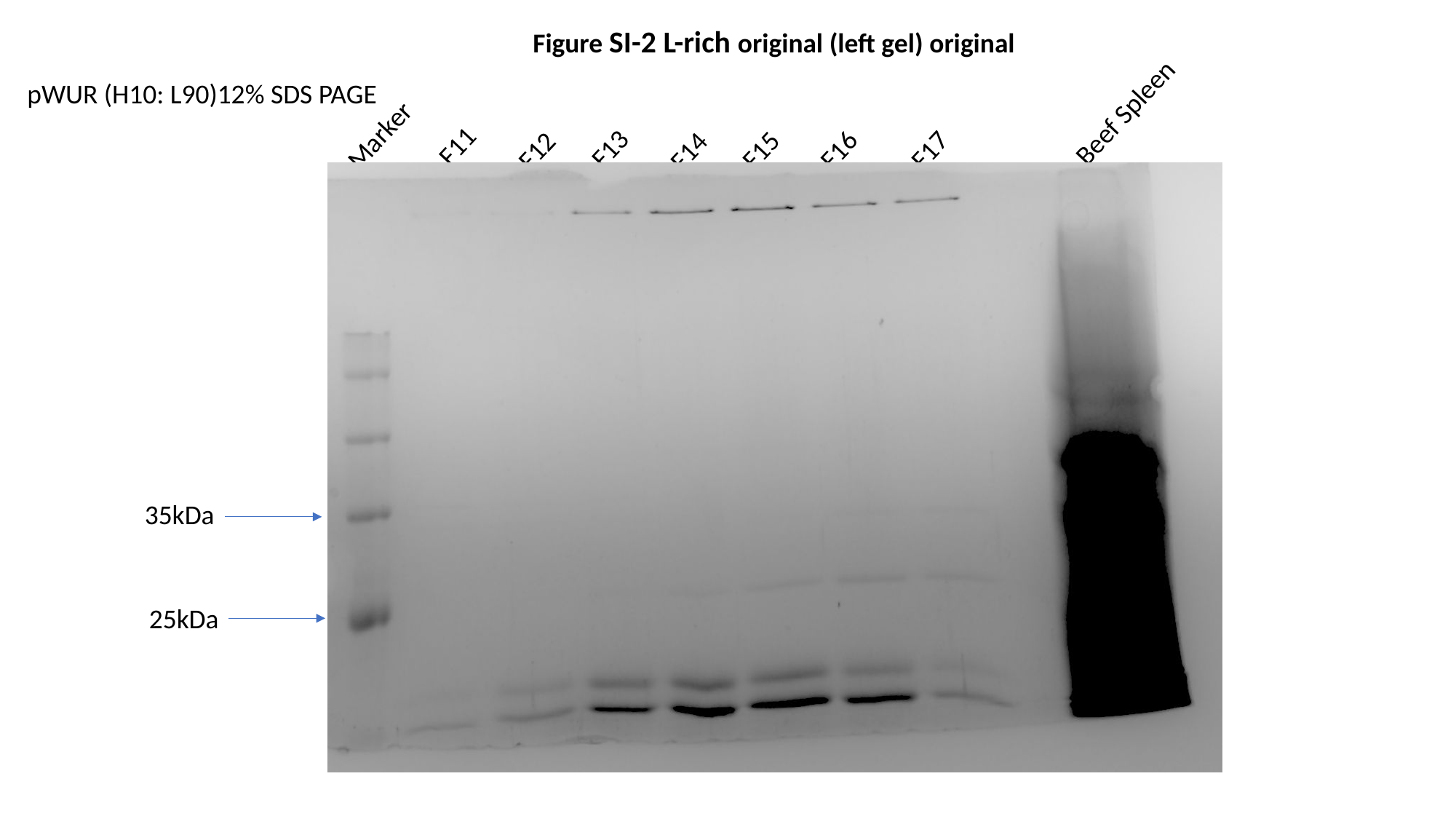

Figure SI-2 L-rich original (left gel) original
pWUR (H10: L90)12% SDS PAGE
Beef Spleen
Marker
F11
F13
F17
F16
F15
F12
F14
35kDa
25kDa

## Slide 30
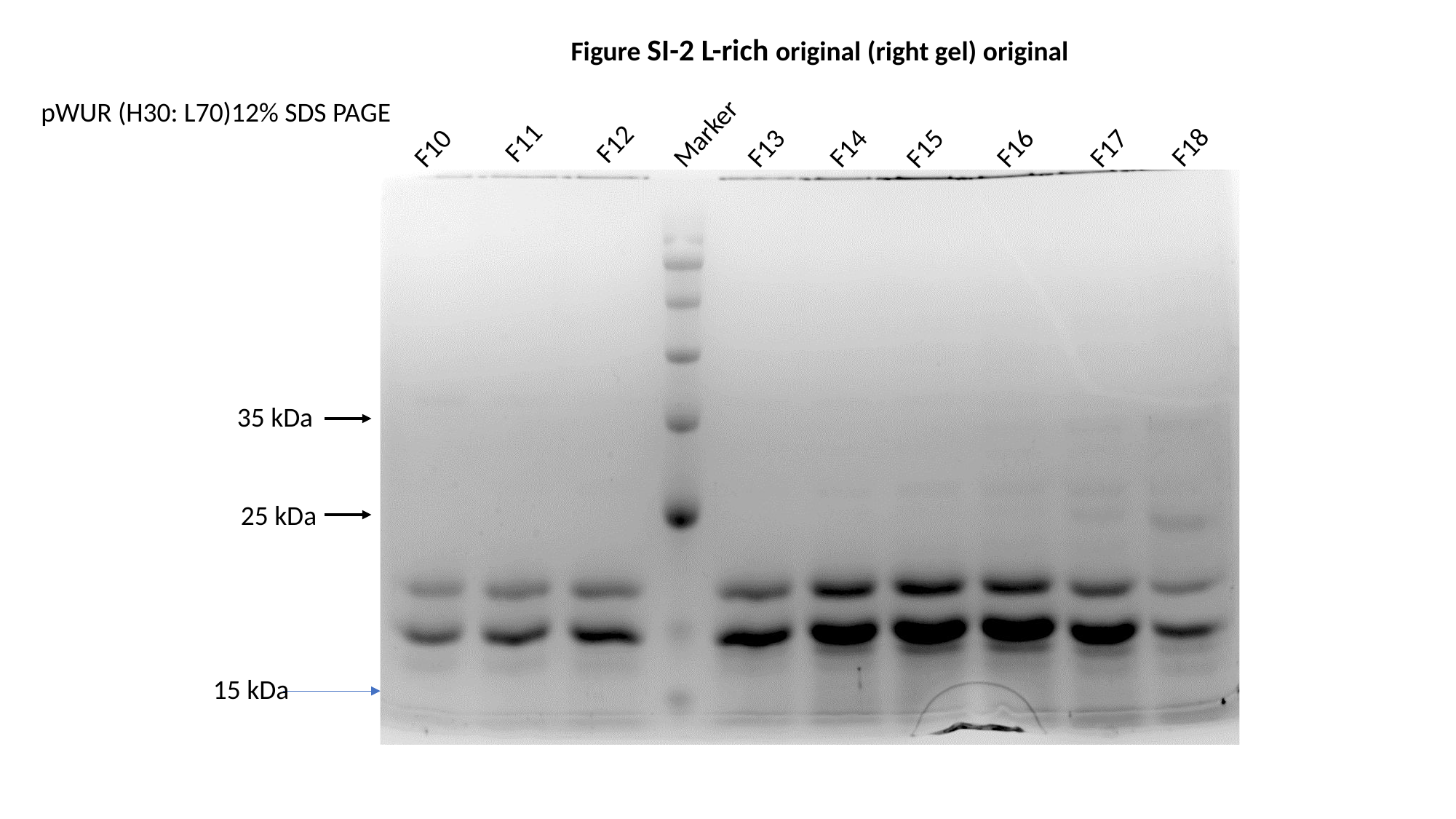

Figure SI-2 L-rich original (right gel) original
pWUR (H30: L70)12% SDS PAGE
Marker
F11
F12
F18
F15
F13
F16
F17
F10
F14
35 kDa
25 kDa
15 kDa
