# Supplementary material for: Ferritin microheterogeneity, subunit composition, functional, and physiological implications
Source: Sci Rep. 2023 Nov 14;13:19862. doi: 10.1038/s41598-023-46880-9 (PMC10646083; doi:10.1038/s41598-023-46880-9)
Supplement: Supplementary file 2 — Supplementary Information 2. [file 41598_2023_46880_MOESM2_ESM.pdf]

Supplementary Figures:

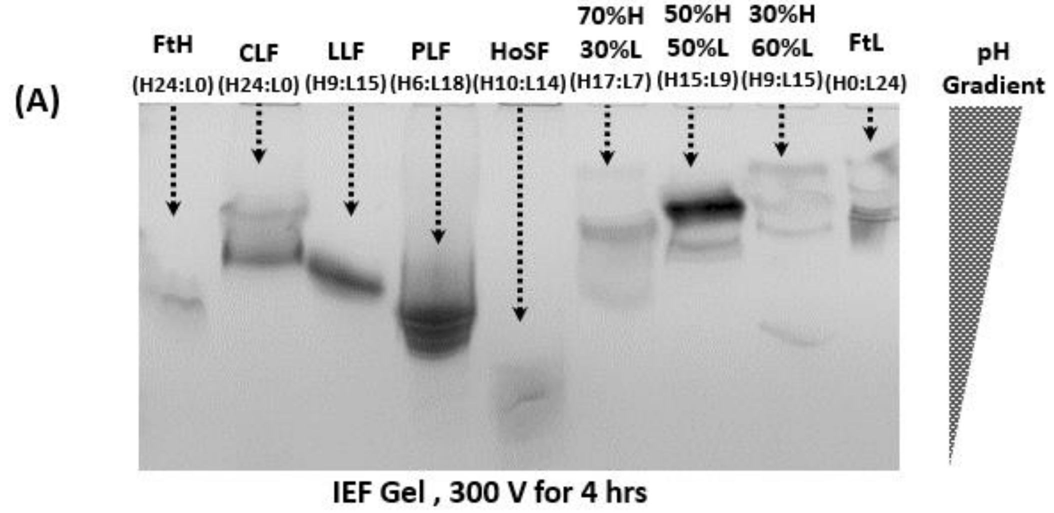

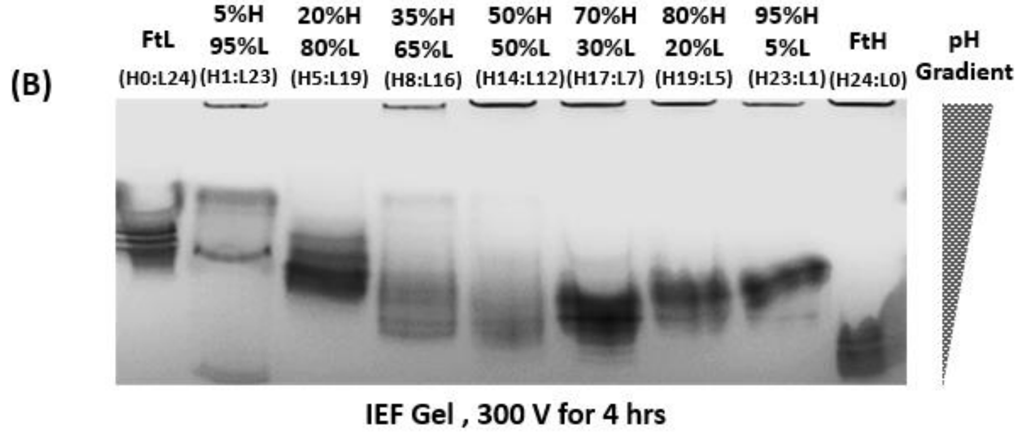

Figure SI-1. Recombinant human homopolymer H (FtH) and L (FtL) and heteropolymer ferritins, and organ extracted ferritins were loaded on Invitrogen Novex native-PAGE isoelectric focusing (IEF) gels (Thermo Fisher Scientific Inc.) for 4hrs at 300V then stained with Coomassie blue. Protein concentrations (0.5-1 µg per well) were purified by size exclusion chromatography before the IEF run.

H-rich Samples

Recombinant Human H-rich Ferritin (~90%H:10%L)

Recombinant Human H-rich Ferritin (~80%H:20%L)

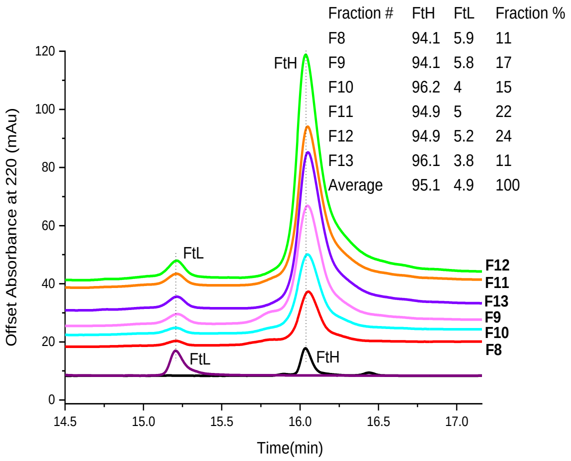

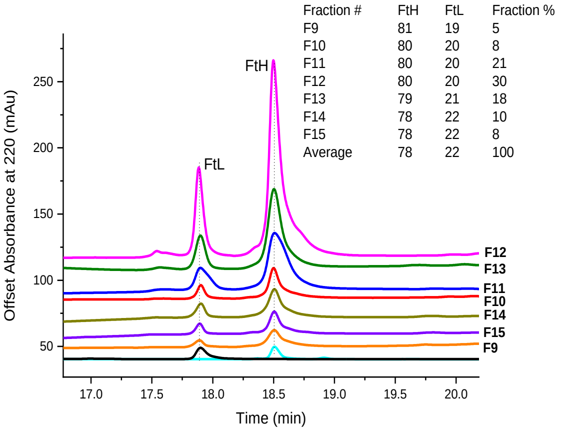

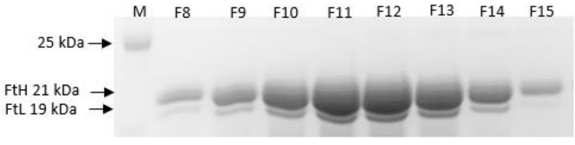

7% Native-PAGE

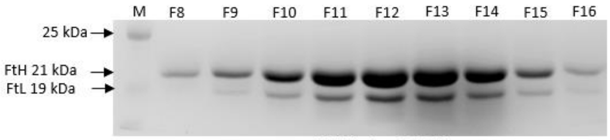

7% Native-PAGE

L-rich Samples

Recombinant Human L-rich Ferritin (~10%H:90%L)

Recombinant Human L-rich Ferritin (~30%H:70%L)

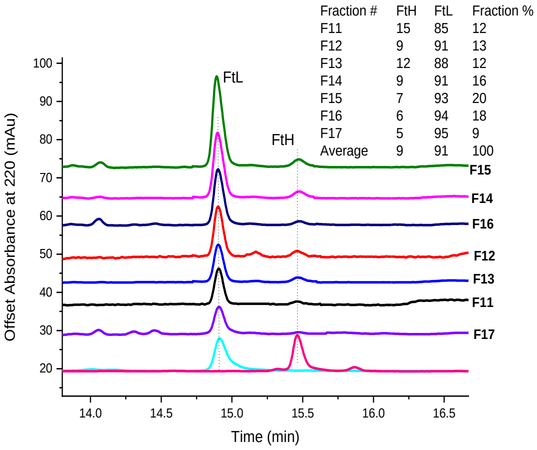

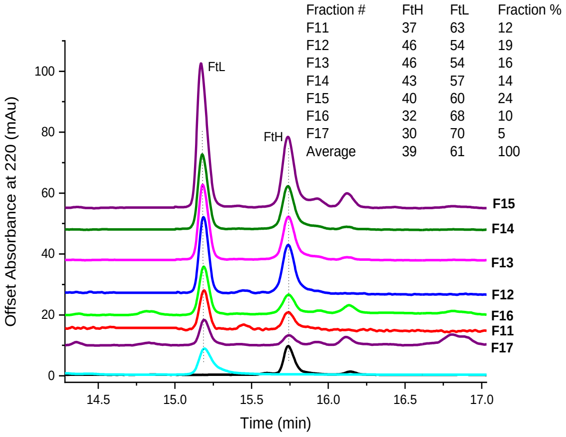

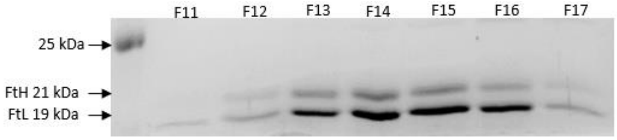

7% Native-PAGE

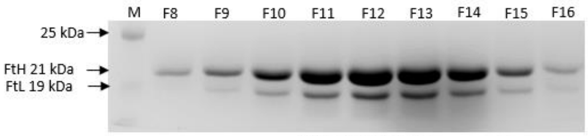

7% Native-PAGE

Figure SI-2: Representative SDS-CGE electropherograms of SEC-fractionated samples of recombinant human heteropolymer L-rich and H-rich ferritins following size exclusion chromatography and the corresponding 7% Native-PAGE of the collected fractions.
